# Nanotechnology‐Fortified Manipulation of Cell Ca^2+^ Signaling

**DOI:** 10.1002/smsc.202400169

**Published:** 2024-06-26

**Authors:** Yaofeng Zhou, Zherui Zhang, Chen Zhou, Yuanhong Ma, Haoye Huang, Junqiu Liu, Dingcheng Zhu

**Affiliations:** ^1^ College of Material, Chemistry and Chemical Engineering Key Laboratory of Organosilicon Chemistry and Material Technology, Ministry of Education Hangzhou Normal University Hangzhou 311121 China; ^2^ School of Engineering Westlake University Shilongshan Road Hangzhou 310030 Zhejiang China; ^3^ College of Chemistry and Chemical Engineering Central South University Changsha 410083 China

**Keywords:** biomedical applications, Ca^2+^ channels, Ca^2+^ signaling manipulation, nanotransducers, working principle

## Abstract

The manipulation of cytosolic Ca^2+^ concentration ([Ca^2+^]_i_) plays a crucial role in the study of Ca^2+^ signaling and the therapy of its affected diseases. Nanotechnology enables the development of nanotransducers for targeted, non‐invasive, highly spatiotemporal, and on‐demand [Ca^2+^]_i_ regulation by responding to external energy fields to activate Ca^2+^ channels, in situ deliver Ca^2+^, or release the payload of chemical modulators. As considerable strides have been made in Ca^2+^ signaling‐related fundamental research and applications in recent years, in this article, it is tried to present a thorough review of nanotransducer‐based [Ca^2+^]_i_ manipulation, from the working principle to specific applications. Focusing on the design rationale and constructions of nanotransducers, the interactions between nanotransducers and Ca^2+^ channels are highlighted, as well as the downstream effectors of Ca^2+^ signaling pathways, followed by their representative biomedical applications in disease treatment and neuromodulation. Moreover, despite the enormous progress made to date, nanotransducer‐regulated Ca^2+^ signaling still confronts obstacles, and several scientific issues urgently need to be resolved. Thus, to provide brief and valid instructions for the development of nanotransducers for the regulation of Ca^2+^ signaling, proposals on how to improve the nanotransducer‐based [Ca^2+^]_i_ manipulation as well as future challenges and prospects are discussed.

## Graphical abstract

This review explores the design rationale and constructions of nanotransducers, and highlights their interactions with Ca^2+^ channels, as well as the downstream effectors of Ca^2+^ signaling pathways. It also discusses various biomedical applications in disease treatment and neuromodulation, and limitations in current approaches, while envisioning future advancements in the development of new generations of safe, efficient, and controllable nanotransducers.

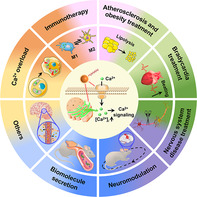

## 1 Introduction

**Scheme 1:**
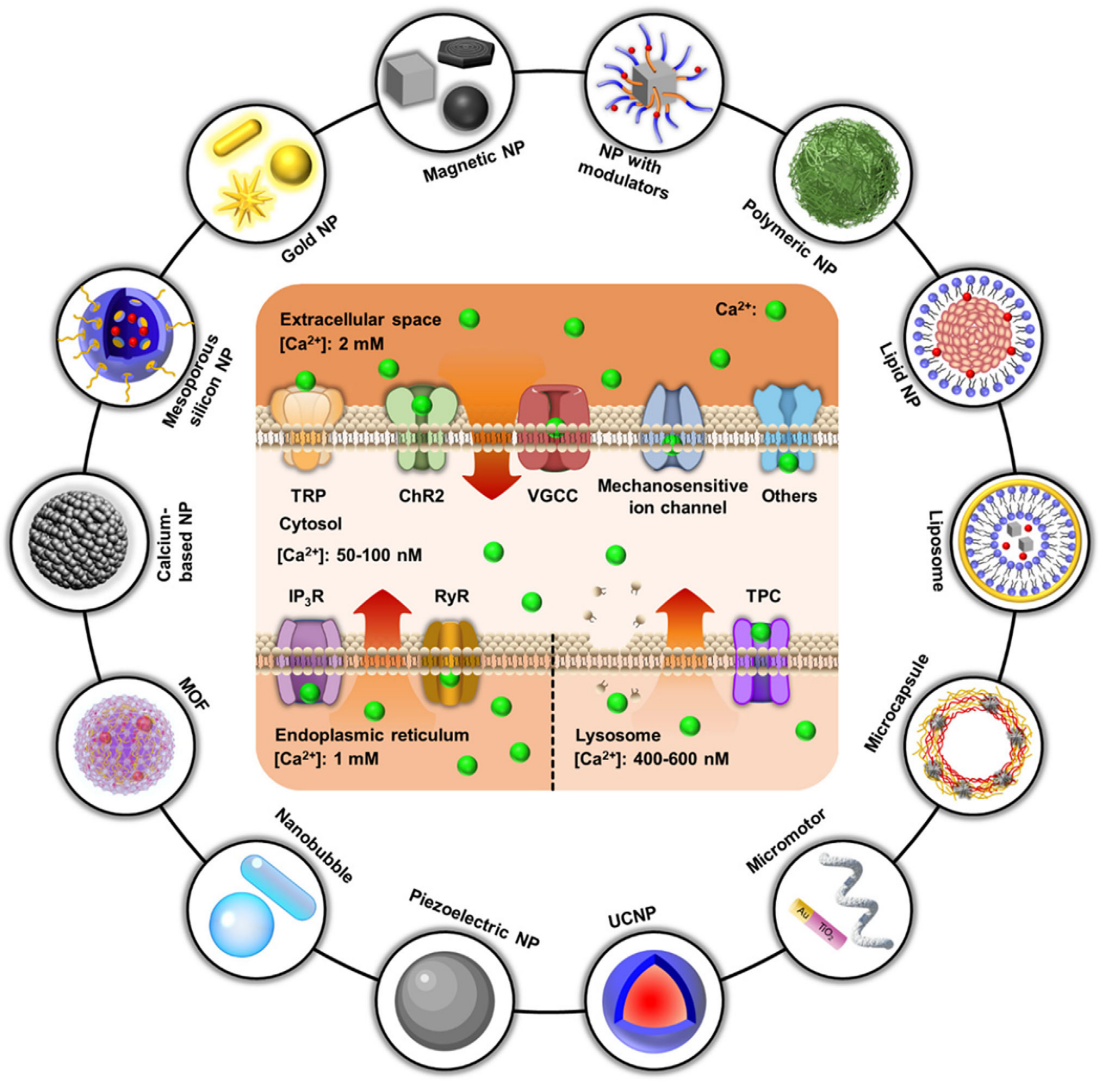
The utilization of nanotransducers for [Ca^2+^]_i_ manipulation. A variety of nanomaterials owing to their unique physicochemical properties have been employed to regulate [Ca^2+^]_i_ by activating Ca^2+^ channels. Under stimulation, [Ca^2+^]_i_ is elevated as a result of extracellular Ca^2+^ influx or endogenous Ca^2+^ release. TRP: transient receptor potential channels. ChR2: channelrhodopsin‐2. VGSC: voltage‐gated sodium channels. VGCC: voltage‐gated calcium channels. IP_3_R: IP_3_ (inositol 1,4,5‐trisphosphate) receptor. RyR: ryanodine receptor. TPC: two‐pore channel. MOF: metal‐organic framework.

Calcium ion (Ca^2+^), a ubiquitous second messenger, regulates diverse physiological processes, such as cell proliferation and apoptosis, fertilization, immunity, skeletal and cardiac muscle contraction, neural transmission at excitatory synapses, and hormone secretion.^[^ ^1^ ^]^ Ca^2+^ signaling modulates physiological processes through an elevation in cytosolic Ca^2+^ concentration ([Ca^2+^]_i_), which is dynamically regulated through extracellular Ca^2+^ influx or endogenous Ca^2+^ release due to the substantial concentration gradients (**Scheme** **1**). Importantly, these [Ca^2+^]_i_ regulatory pathways could be activated by multiple external stimuli, allowing for the artificial manipulation of Ca^2+^ signaling. Moreover, Ca^2+^ transients within a localized region can spread throughout the entire cell in the form of intracellular Ca^2+^ waves and may further propagate to neighboring cells to trigger intercellular Ca^2+^ waves for an organ‐level integrative response.^[^ ^2^ ^]^ This means that local manipulation of [Ca^2+^]_i_ could be developed as a virtuous tool for investigating Ca^2+^ signaling and the related physiological processes.

Being inspired, several strategies such as chemical regulation, electrical stimulation, optogenetics, and focused ultrasound manipulation, have been developed to trigger a Ca^2+^ response in vivo. Strategies based on Ca^2+^ channel control by introducing external stimuli have significantly accelerated the study of Ca^2+^‐dependent biological processes, control of neuronal activity, and the treatment of Ca^2+^ dysregulation‐associated diseases. Nonetheless, these conventional approaches confront arduous challenges as a result of the growing demands for in‐depth mechanism investigation and application expansion. Chemical regulation relies on the delivery of chemical modulators to target ion channels and is restricted by insufficient payload efficiency, potential off‐target effect, and low temporal resolution with a delayed response time ranging from minutes to hours.^[^ ^3^ ^]^ Tethering an optical fiber or electrical wire to a patient for chronic deep brain stimulation is highly invasive and frequently carries the risk of hardware complications, such as tissue damage, infection, the immune response at the implant‐tissue interface, and implant fracture.^[^ ^4^ ^]^ Focused ultrasound is severely constrained by its lack of spatial and temporal specificity, nonstationarity, and the risk of heating.^[^ ^5^ ^]^ Collectively, the development of reliable tools with targetability, high spatiotemporal precision, remote control, and minimal invasiveness for manipulating cell Ca^2+^ signaling is highly desirable.

Several innovative techniques, such as non‐invasive alternating current stimulation, temporal interference stimulation, and nanotransducer‐mediated stimulation, have been developed to address the limitations of conventional methods.^[^ ^6^, ^7^, ^8^ ^]^ Among them, nanotransducers are particularly attractive in terms of tunable physicochemical properties, structures and functions, and the potential for large‐scale manufacture. It is worth mentioning that [Ca^2+^]_i_ manipulation by nanotechnology could be accomplished through minimally invasive injection. Nowadays, various nanomaterials, such as photothermal nanoparticles (NPs), magnetic NPs, piezoelectric NPs, up‐conversion NPs (UCNPs), and calcium‐based nanomaterials, which can respond rapidly and specifically to external stimuli have been utilized to develop alternative approaches for eliciting Ca^2+^ response in living cells. The nanoplatforms with tunable size effect, cell‐nano interfaces, optical properties, and catalytic activities can enhance their [Ca^2+^]_i_ regulation efficiency.^[^ ^9^ ^]^ Nanomaterials with energy conversion properties have been devised to rapidly transform macroscopic inputs that deeply penetrate tissue, such as near‐infrared (NIR) light,^[^ ^10^, ^11^ ^]^ magnetic fields,^[^ ^12^, ^13^ ^]^ and ultrasound,^[^ ^14^, ^15^ ^]^ into one or more local stimuli for modulating specific Ca^2+^ channels at the level of a single cell, thereby enabling remote control of cellular functions and activities. Utilizing nanotransducers to deliver chemical stimuli for modulating Ca^2+^ dynamics increases payload efficiency, the bioavailability, and reduces adverse effects. Among those, calcium‐based nanomaterials enable direct delivery of Ca^2+^ to target cells, and nanomaterials with catalytic properties can in situ generate chemical stimuli. The development of nanotransducers with high efficiency, excellent spatiotemporal resolution, and minimally invasive modulation of [Ca^2+^]_i_ for studying and utilizing Ca^2+^‐regulated physiological processes, has aroused widespread interest, paving the way for advances in fundamental research and clinical translation.

Given the crucial role of Ca^2+^ in the regulation of cellular processes and disease treatment, several excellent review articles on the development of nanotransducers for [Ca^2+^]_i_ manipulation have been recently published. We notice that most current review papers focused on elucidating a single type of nanotransducers, such as magnetic nanoswitches,^[^ ^12^, ^13^ ^]^ piezoelectric nanomaterials,^[^ ^14^, ^15^ ^]^ and light‐responsive nanoregulators,^[^ ^10^, ^11^ ^]^ or one certain downstream application, such as neuromodulation,^[^ ^9^, ^16^ ^]^ theragnostic,^[^ ^17^ ^]^ and tumor therapy.^[^ ^18^, ^19^ ^]^ It is noteworthy that numerous novel nanotransducers capable of generating local mechanical force, upconverted or focused light, ultrasound, and chemical stimulators, have emerged for precise activation of Ca^2+^ signaling. In addition, activation of cell Ca^2+^ signaling has also been successfully implemented in many new fields, including regulation of the immune system, treatment of atherosclerosis and obesity, alleviation of neurodegenerative disorders, control of animal behaviors, initiation of stem cell differentiation, and transient opening of the blood–brain barrier (BBB). To the best of our knowledge, a comprehensive review covering the most recent advancements in the development of nanotransducers, from the design philosophy of emerging technologies to their novel application scenarios, is still lacking. Thus, we aim to summarize the most recent emerging strategies systematically and thoroughly for utilizing nanotransducers to wirelessly trigger Ca^2+^ responses, including nanotransducer‐generated physical stimuli and nanotransducer‐enhanced payload of chemical modulators. Meanwhile, this article emphasizes the target Ca^2+^ channels, their interactions with nanotransducers, and the mechanisms that govern Ca^2+^ responses triggered by nanotransducers (Scheme 1). Subsequently, important advances in nanotransducer‐based biological applications, such as Ca^2+^ overload‐based cancer therapy, treatment of atherosclerosis, obesity, bradycardia, and nervous system disease, controlling hormone secretion, as well as the improvement of nanomaterials for the advancement of these applications, are demonstrated. Finally, this review concludes with a discussion of the existing challenges and further improvements for the development of a new generation of nanotransducers and their potential future applications.

## 2 Basic Working Principles of Nanotransducers for Regulating [Ca^2+^]_i_

**Scheme 2:**
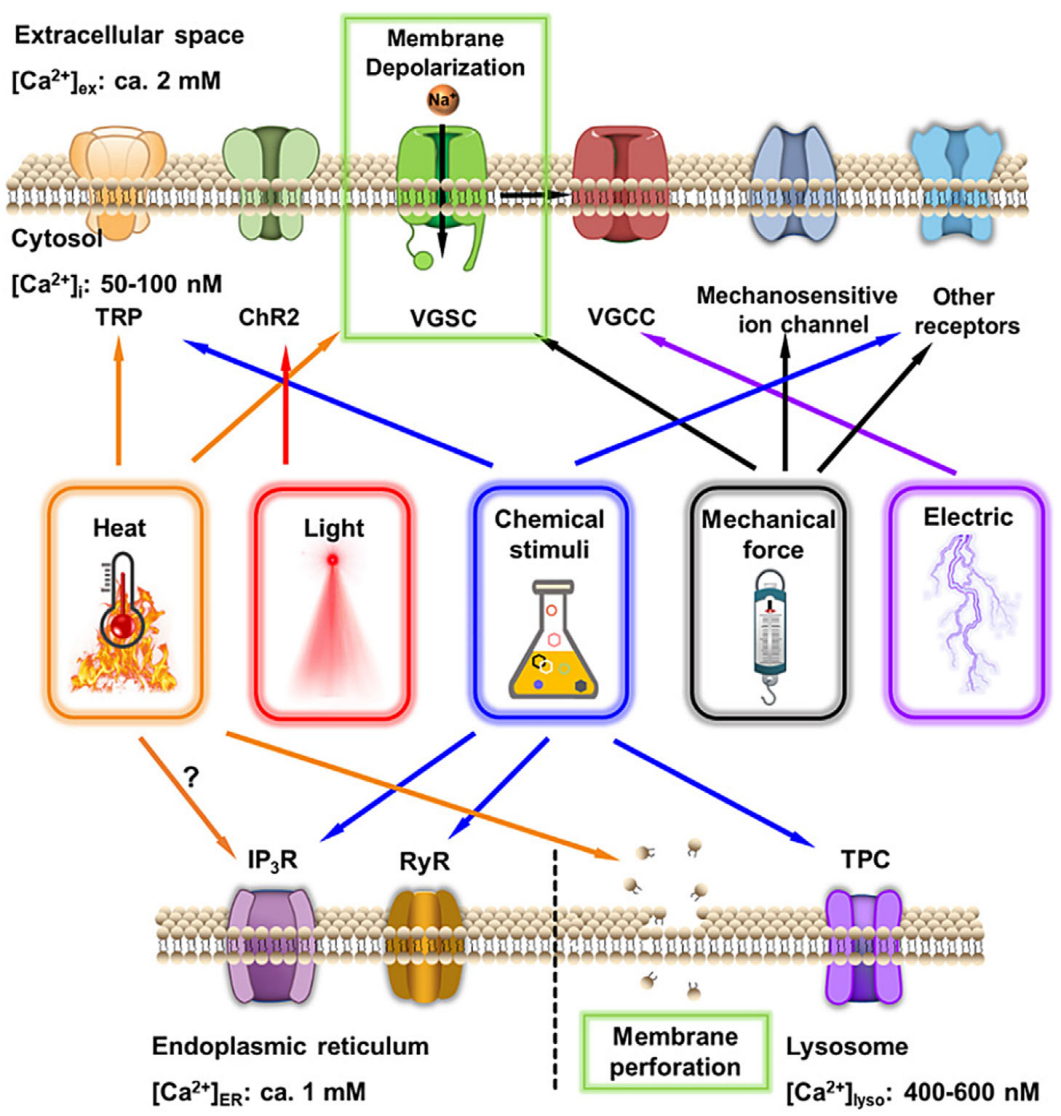
Nanotransducers could generate a series of signals including heat, light, mechanical force, electrical, and chemical stimuli to activate Ca^2+^ channels.

**Scheme 3:**
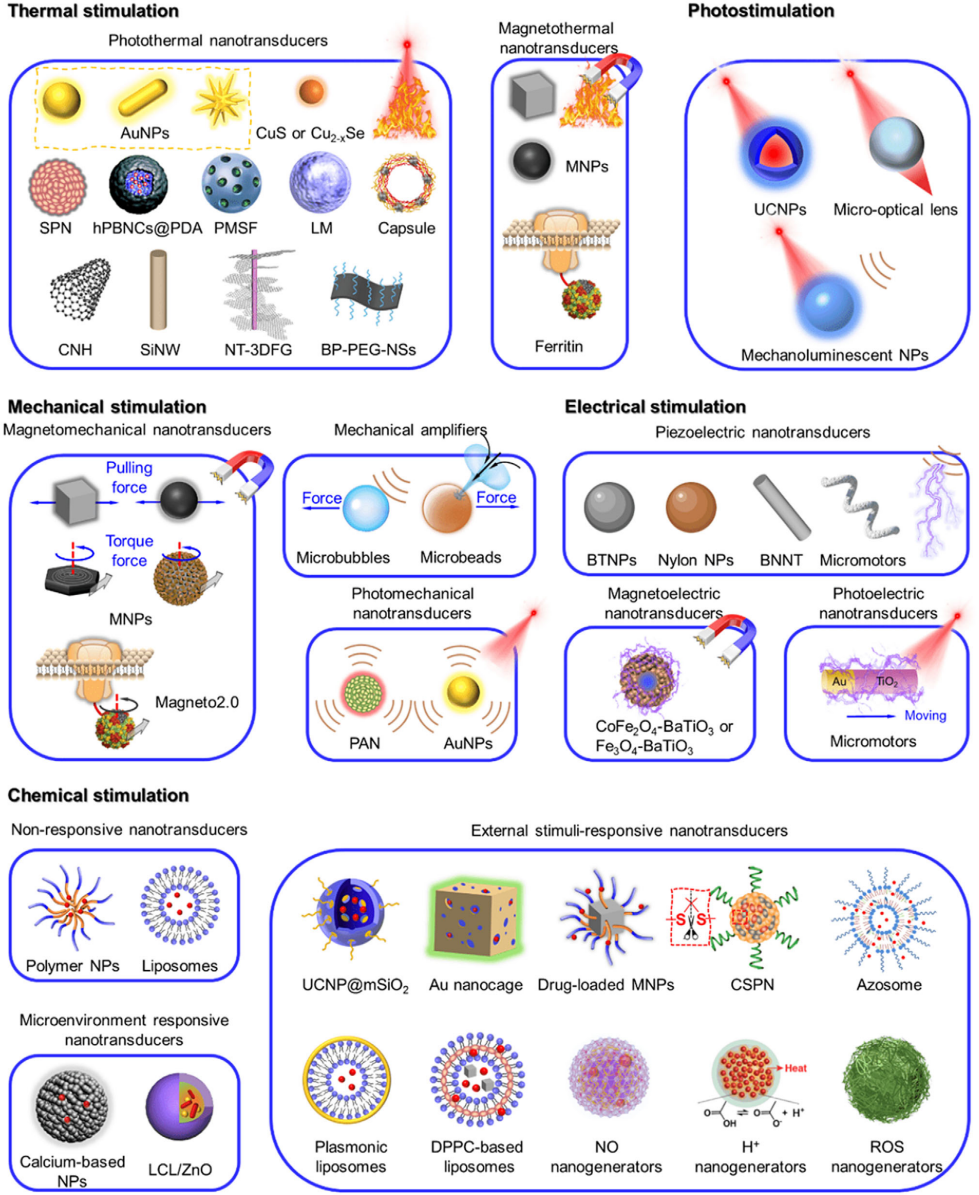
A summary of the recently reported nanotransducers and their working principles of manipulating [Ca^2+^]_i_. In the presence of external energy fields, nanotransducers transform the input energy to heat, optical, mechanical force, electrical, and chemical signals to induce an increase in [Ca^2+^]_i_.

The introduction of nanotechnology to manipulate [Ca^2+^]_i_ was originally proposed to improve the pharmacokinetics and toxicity profiles of chemical modulators.^[^ ^20^ ^]^ The capacity to concentrate chemical stimuli on Ca^2+^ channels of target cells is critical for maximizing stimulation efficiency and preventing off‐target effects. With the rapid development of nanotechnology and increased knowledge of activating principles for Ca^2+^ signaling, nanomaterials have also been engineered to convert external energy into local signals within specific regions of target cells. Particularly, genetically encoded exogenous Ca^2+^ channels broaden the applicability of [Ca^2+^]_i_ manipulation in diverse cell types. The general work principle is shown in **Scheme** **2**. By converting external input energy into physical signals or delivering/generating chemical stimuli, nanotransducers could efficiently stimulate specific Ca^2+^ channels in target cells. Given the significant efforts invested in the development of nanotransducers, this section provides for the first time a detailed overview of the design rationale, construction methods, and working principles. Herein, output signals of nanotransducers for manipulating [Ca^2+^]_i_ are classified into thermal stimulation, photo modulation, mechanical regulation, electrical manipulation, and chemical activation. Representative examples are elaborately described aiming at presenting the advancement of this field and providing paths for further spark collision research. **Table** **1** provides a summary of commonly activated Ca^2+^ channels and their related stimulatory signals, which are the basis for manipulating [Ca^2+^]_i_ with nanotransducers. The manipulation pathways of most nanotransducers are presented in Scheme 2 to provide a rapid index channel. **Scheme** **3** summarizes the majority of nanotransducers mentioned in this review paper and their working principles of manipulating [Ca^2+^]_i_.

**Table 1:** The summary of working principles and biomedical applications of Ca^2+^ signaling nanotransducers.

Input signal	Stimuli	Temporal response	Manipulation principle	Applications	Ref.
Light	Heat	≈ms	Gold nanorods labeled with anti‐TRPV1 antibody could specifically bond with TRPV1 on cancer cells. Then, the gold nanorods could perform a photothermal effect to stimulate TRPV1 and evoke Ca^2+^ influx.	Nanotransducers evoked Ca^2+^ influx for Ca^2+^ overload cancer therapy.	[164]
Light	Heat	≈ms	Gold NPs targeting TRPV1 or P2X3 of DRG neurons and triggering membrane depolarization through photothermal effect.	Nanotransducers triggered Ca^2+^ response and therefore achieved neuron activation.	[28]
Light	Heat	N/A	Gold nanorods bond to TRPV1, integrins of TRPV1^+^‐HEK293T cells, and unmodified HEK293 cells. Then, the photothermal effect of gold nanorod could activate TRPV1 or endoplasmic reticulum for increasing [Ca^2+^]_i_.	Nanotransducers‐induced Ca^2+^ response could regulate the expression of eGFP.	[224]
Light	Heat	N/A	CuS nanoparticle decorated with anti‐TRPV1 antibody could target with TRPV1 of vascular smooth muscle cells and evoke Ca^2+^ influx by photothermal effect under the NIR irradiation.	Nanotransducers‐activated Ca^2+^ signaling and attenuated atherosclerosis.	[29]
Light	Heat	N/A	Ca^2+^ influx was triggered by using Cu_2−*x*_Se NPs to target and activate TRPV1 of BV2 cells and stimulate TRPV1 by the NIR‐induced photothermal effect of Cu_2−*x*_Se NPs.	Nanotransducers‐activated Ca^2+^ signals could promote phagocytosis.	[30]
Light	Heat	0.9 s for cells and 5.0 s for mouse	A semiconducting polymer core and an amphiphilic polymer shell formed nanotransducer could activate TRPV1 of TRPV1^+^‐HEK293T cells and mice by photothermal effect under NIR II irradiation for Ca^2+^ influx.	Nanotransducers‐evoked Ca^2+^ response was observed to control the behaviors of mice.	[37]
Light	Heat	≈ms	PEGylated carbon nanohorn was considered to activate TRPV2 on the TRPV2‐expressed cancer cells through photothermal effect under NIR II irradiation for Ca^2+^ influx.	Ca^2+^ influx‐induced nanotransducers could kill cancer cells by Ca^2+^ overload‐caused mitochondria dysfunction.	[165]
Light	Heat	≈ms	Nanowires‐templated 3D graphene enabled the stimulation of membrane depolarization with lower laser energies and therefore induced Ca^2+^ influx.	Ca^2+^ influx could be used for cell signaling research.	[47]
Light	Heat	≈ms	AuNPs performed a photothermal effect to depolarize the cell membrane of hippocampal neurons to induce Ca^2+^ influx.	The evoked Ca^2+^ influx could be used to activate neurons.	[49]
Light	Heat	≈ms	Polyelectrolyte capsules combined with gold nanostars could achieve photothermally controlled Ca^2+^ release from the lysosome, and meanwhile increased [Ca^2+^]_i_ of cancer cells through CICR.	This strategy could be used to investigate the underlying mechanism of CICR.	[50]
Light	Heat	N/A	Silicon nanowires with photothermal function could trigger internal Ca^2+^ storage organelles to release Ca^2+^ in Glia cells, U2OS, and HUVEC cells.	The proposed method is a potential tool for Ca^2+^ manipulation.	[53]
Light and acidity	Heat and Ca^2+^ release	N/A	CuS‐loaded CaCO_3_ nanoparticles could trigger Ca^2+^ influx by tumor acidic microenvironment degraded CaCO_3_ nanoparticle and CuS performed photothermal stimulation.	The increased [Ca^2+^]_i_ induced the dysfunction of mitochondria of cancer cells for cancer therapy.	[39]
Light	Light (450 and 470 nm)	≈ms	UCNPs converted NIR light to 450 and 470 nm light to stimulate ChR2 on HeLa cells and evoke Ca^2+^ influx.	Nanotransducers could be used to research Ca^2+^ signaling.	[60]
Light	Light (456 and 470 nm)	≈ms	UCNPs targeting glycoconjugate of ChR2^+^‐HEK293 cells and convert NIR light to blue light to activate ChR2 to induce Ca^2+^ influx.	The evoked [Ca^2+^]_i_ could induce cell apoptosis of cancer cells thereby achieving cancer therapy.	[65]
Light	Light 470 nm	≈s	UCNPs activated the CRAC channel of HeLa cells and BMDCs and induced Ca^2+^ influx.	Ca^2+^ manipulation was used for immunity modulation.	[184]
Light and ultrasound	Light 470 nm	≈ms	ZnS:Ag,Co@ZnS NPs could respond to light and ultrasound irradiation to activate ChR2 on the HEK293 cell membrane and then accelerate Ca^2+^ influx.	Ca^2+^ manipulation was used to modulate the behaviors of mice.	[67]
Light	IP_3_	≈ms	Photosensitive nanocapsule was used to deliver IP_3_ to stimulate IP_3_R of CHO‐MI cells for releasing Ca^2+^ from ER.	A potential strategy for Ca^2+^ signal research.	[139]
Light	NO	N/A	ZIF‐8 was used to encapsulate NO donors on the surface of UCNPs. Upon NIR light irradiation, the blue light could degrade the S‐NO bond and release NO to stimulate RyRs of 4T1 cells.	Increased [Ca^2+^]_i_ affected the function of mitochondria and induced cancer cell apoptosis.	[172]
Light	ROS	N/A	NIR‐responsive oligomer was used to prepare a water‐soluble NP and generate ROS under NIR irradiation to stimulate TRPM2. The activated TRPM2 on the cancer cell membrane facilitated the Ca^2+^ influx.	The method of increasing [Ca^2+^]_i_ was used to treat cancer.	[150]
Light	ROS and NO	N/A	Mesoporous silica NPs were used to encapsulate ICG and BNN6. ROS were generated under NIR irradiation to stimulate TRPA1. NO was generated under photothermal heating to stimulate RyRs.	The method of increasing [Ca^2+^]_i_ from endogenous sources was used to treat cancer.	[168]
Light	SKF‐81 297	≈s	Photoswitchable nanovesicles responded to 455 nm irradiation to release the encapsulated SKF‐81 297 to activate the dopamine D1‐receptor of primary striatal neurons and increase [Ca^2+^]_i_.	Modulation of neuronal activities.	[143]
Light	H_2_O_2_	≈ms	Photocatalytic MOFs could activate IP_3_Rs and induce Ca^2+^ outflow from ER.	Modulation of neuronal activities and control of animal behaviors.	[152]
AMF	Heat	≈10 s	Fe_3_O_4_ nanoparticles with magnetothermal function under AMF irradiation could stimulate TRPV1 to accelerate Ca^2+^ influx.	Ca^2+^ activation for proinsulin release	[35]
AMF	Heat	2.2 s	Fe_3_O_4_ nanoparticles could target glycosylated cell membrane proteins. Meanwhile, Fe_3_O_4_ nanoparticles respond to AMF to activate TRPV1 of hippocampal neurons for Ca^2+^ influx.	Ca^2+^ influx was used to study mouse behaviors.	[210]
AMF	Heat	≈10 s	Fe_3_O_4_ nanoparticle targeting TRPV1 of mouse adrenal gland to increase [Ca^2+^]_i_ through promoting Ca^2+^ influx.	The increased [Ca^2+^]_i_ could accelerate hormone secretion.	[213]
AMF	Heat	≈5 s	To manipulate [Ca^2+^]_i_ of TRPV1^+^‐HEK293FT cells, Fe_3_O_4_ nanoparticle was used to target TRPV1 and induced Ca^2+^ influx through magnetothermal effect.	Ca^2+^ influx was used to activate the neuron.	[207]
AMF	Heat	≈s	Fe_3_O_4_ nanoparticles with magnetothermal function could heat the TRPV1 and TRPA1 of DRG and DRG explants for inducing Ca^2+^ influx.	The Ca^2+^ signal was used to modulate axonal growth.	[197]
AMF	H^+^	≈32 s	Magnetic nanoparticles modified by poly(sebacic acid)/poly(lactic‐co‐glycolic acid) for controlling local pH and activating TRPV1 of hippocampal cells.	An approach for Ca^2+^ signaling research.	[225]
AMF	Dopamine	≈s	Magnetic nanoparticles cooperated with polymer to release dopamine to activate D_1_ and D_2_ receptors of striatal neurons with increased [Ca^2+^]_i_.	Activating neuron.	[144]
Magnetic field	Pulling force (≈0.1 pN)	≈ms	Cubic Zn_0.4_Fe_2.6_O_4_ nanoparticles could target glycoconjugate on the inner ear hair cell membrane of *Rana catesbeiana*. Under magnetic field control, the nanotransducer generates a pulling force to activate mechanosensitive ion channels and induce Ca^2+^ influx.	Neuron activation.	[78]
Magnetic field	Pulling force (200–350 pN)	≈s	Starch‐coated magnetic nanoparticles generated a pulling force under the control of a magnetic field to activate the N‐type Ca^2+^ channel of cortical neurons.	This approach was used to modulate endogenous ion channel expression.	[83]
Magnetic field	Torque force	≈s	Fe_3_O_4_ nanodiscs respond to the magnetic field to generate torque force to activate TRPV4 and Piezo2 of DRG and hippocampal neurons. The activated ion channels accelerated the Ca^2+^ influx.	This nanotransducer could be used for Ca^2+^ signaling study.	[85]
Ultrasound	Mechanical force	≈s	PS microbeads bound to integrin and stimulated P2X of HEK293T cells and manipulated [Ca^2+^]_i_.	It is a potential nanotransducer for Ca^2+^ signaling study.	[92]
Ultrasound	Mechanical force	≈s	Microbubbles targeting integrin were used to stimulate Piezo1 of HEK293, Jurkat T, and PBMC cells and meanwhile manipulate [Ca^2+^]_i_.	This method was used to activate the chimeric antigen receptor.	[167]
Ultrasound	Electrical stimulation	≈s	BT NPs could respond to ultrasound signals to generate an electrical signal and stimulate VGCC of SH‐SY5Y cells. The activated VGCC increased the [Ca^2+^]_i_.	Piezoelectric nanomaterials could be used to study the electronic signal on manipulating [Ca^2+^]_i_.	[103]
Ultrasound	Electrical stimulation	≈s	C@BT NP could respond to ultrasound to generate an electronic signal to stimulate the L‐type Ca^2+^ channel of PC12 cells.	The Ca^2+^ manipulation was used to treat Parkinson's disease.	[104]
Light	Electrical stimulation	–ms	The electron‐rich TiO_2_(e^−^) of non‐Faradaic ZST could depolarize the membrane and cause a Ca^2+^ response in cortical neurons.	The Ca^2+^ manipulation was used to control animal behaviors and treat Parkinson's disease.	[118]
Ultrasound	Mechanical force	≈s	PS microbeads targeted integrin and activated TRPM7 of HeLa cells, therefore, increasing [Ca^2+^]_i_.	A potential method for studying Ca^2+^ signaling.	[96]
Ultrasound	Mechanical force	≈ms	Nano gas vesicles can repeatedly activate mechanosensitive cation channels for Ca^2+^ influx.	A potential method for neurostimulation with low‐intensity ultrasound.	[94]
Acidity	Capsaicin	N/A	CaCO_3_ nanoparticle encapsulated with capsaicin. The nanosystem could be degraded by acidity to release capsaicin for activating TRPV1.	The increased [Ca^2+^]_i_ could induce cancer cells apoptosis for cancer therapy.	[133]
N/A	NAADP	≈min	Liposomes contained with NAADP could release t in cells to trigger intracellular Ca^2+^ store for the liberation of Ca^2+^.	This nanotransducer is a promising tool to promote revascularization.	[123]

### 2.1 Thermal Stimulation

#### 2.1.1 Heating‐Induced Ca^2+^ Influx

Transient receptor potential vanilloid1 (TRPV1), one of the most extensively studied Ca^2+^ permeable channels located on the cell surface, has been observed to respond within milliseconds to a variety of environmental and chemical stimuli, such as heat (>43 °C),^[^ ^21^ ^]^ mild acidity (pH < 5.2),^[^ ^22^ ^]^ capsaicin,^[^ ^23^ ^]^ allyl isothiocyanate,^[^ ^24^ ^]^ hydrogen peroxide (H_2_O_2_),^[^ ^25^ ^]^ nitric oxide (NO),^[^ ^26^ ^]^ etc. Mammals express TRPV1 heterogeneously in the primary sensory nervous system, microglia, astrocytes, skin cells, muscle cells, adipose tissue, immune cells, and some types of tumors.^[^ ^27^ ^]^ Hence, TRPV1 has been considered a potential target of nanotransducer‐enabled activation for the investigation of Ca^2+^‐related physiological processes and disease treatment. Indeed, a large number of nanotransducers have been designed to activate TRPV1 by delivering physical and chemical stimuli in situ. Among them, photothermal and magnetothermal nanomaterials featuring excellent energy conversion efficiency, high spatiotemporal resolution, and remote‐controlled stimulation have received tremendous interest, including gold NPs (Au NPs),^[^ ^28^ ^]^ ultrasmall copper sulfide (CuS)^[^ ^29^ ^]^ or copper selenium (Cu_2−*x*_Se) nanodots,^[^ ^30^ ^]^ semiconducting polymer NPs,^[^ ^31^ ^]^ mesoporous silica frameworks hybridized with semiconducting polymer dots,^[^ ^32^ ^]^ carbon nanohorns,^[^ ^33^ ^]^ Prussian blue nanocages coated with polydopamine,^[^ ^34^ ^]^ magnetic NPs (MNPs)^[^ ^35^ ^]^ and genetically engineered ferritin NPs.^[^ ^36^ ^]^

**Figure 1:**
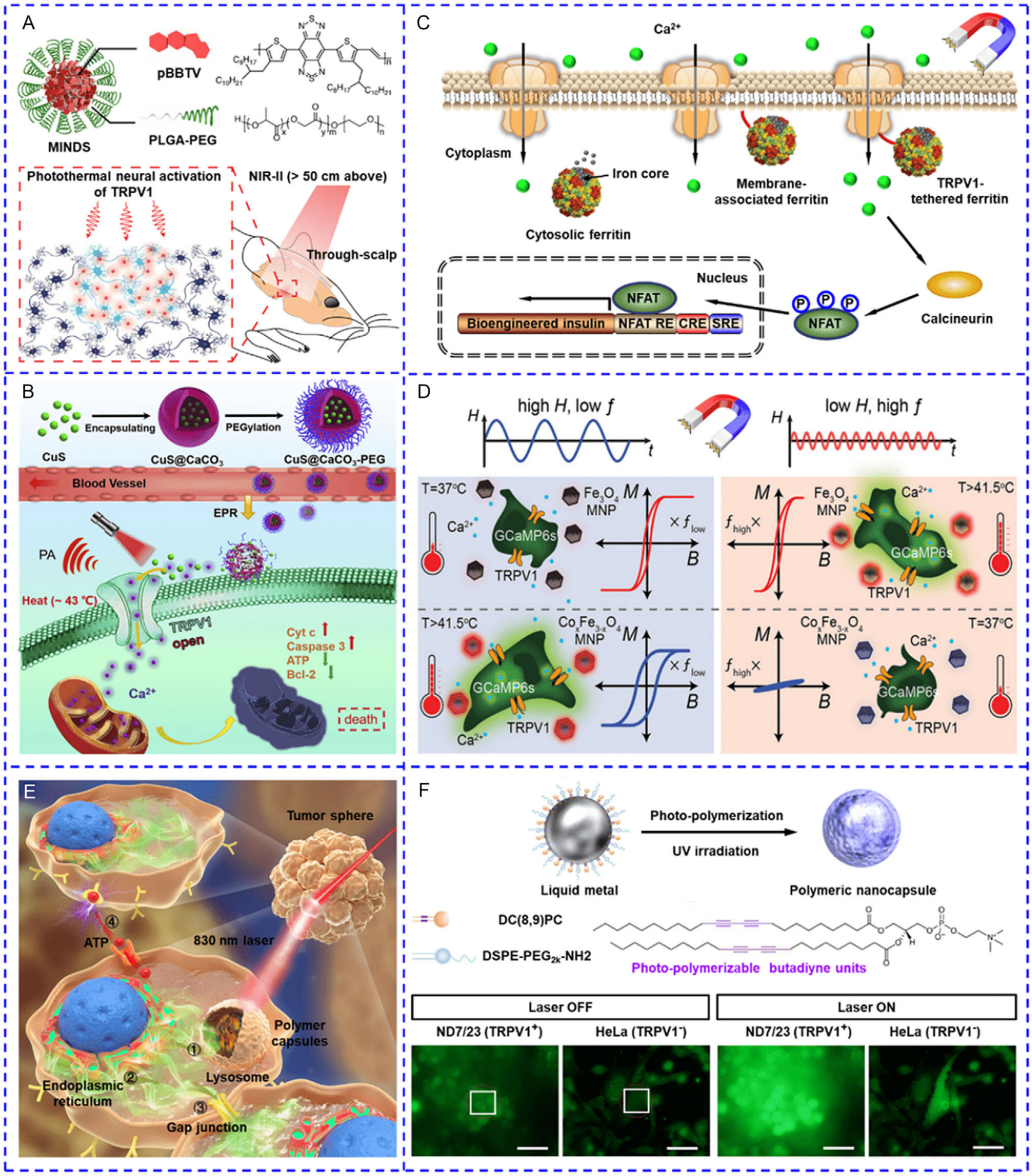
A) Semiconducting polymers coated with PEG‐PLGA shell for performing photothermal effect to activate TRPV1 in the deep brain of freely moving mice. Reproduced with permission.^[^ ^37^ ^]^ Copyright 2022, Springer Nature. B) Working flow for CuS nanodots loaded PEGylated CaCO_3_ acted as a reservoir to supply abundant Ca^2+^ for enhancing TRPV1‐gated Ca^2+^ influx. Reproduced with permission.^[^ ^39^ ^]^ Copyright 2020, Cell Press. C) Schematic diagram of three genetically encoded endogenous paramagnetic iron‐loaded ferritin NPs with varied proximity to TRPV1 channels. Reproduced with permission.^[^ ^42^ ^]^ Copyright 2015, Springer Nature. D) Schematic diagram showing two magnetothermal NPs for multiplexed [Ca^2+^]_i_ regulation. Reproduced with permission.^[^ ^44^ ^]^ Copyright 2020, Wiley. E) Working mechanism of photothermal heating of polymer capsules‐induced propagation of intracellular and intercellular Ca^2+^ wave. Reproduced with permission.^[^ ^50^ ^]^ Copyright 2021, Wiley. F) Photothermal heating of liquid metal NPs evoked Ca^2+^ response in both TRPV1‐expressing and TRVP1‐negative cells. Reproduced with permission.^[^ ^52^ ^]^ Copyright 2017, Springer Nature.

Considering the requirements of practical applications, NIR or, more specifically, NIR‐II windows‐activated photothermal nanotransducers are in significant demand owing to their remarkable tissue penetration, and they have received enormous research interests in the fields of deep brain stimulation and cancer therapy. For instance, Pu's group constructed a photothermal nanoabsorber by coating semiconducting polymer within an amphiphilic polymer shell.^[^ ^37^ ^]^ The capacity of the NIR‐II laser to penetrate deep tissue and the high photothermal conversion efficiency of this photothermal polymer NP (up to 71% at 1064 nm) made this nanotransducer an ideal candidate for tether‐free deep brain stimulation in freely moving mice (**Figure** **1**A). Antibody modification allows for precise targeting of nanotransducers to the cells of interest and offers several advantages, including lowering the administration dosage, increasing the local concentrations of nanotransducers, and reducing off‐target heating, hence minimizing adverse effects in practical applications. Chen et al. modified indocyanine green (ICG)‐loaded polymer micelles with anti‐TRPV1 antibodies so that the nanotransducer could bind to the TRPV1 channel on the membrane of neuronal cells.^[^ ^38^ ^]^ To enhance Ca^2+^ influx‐mediated Ca^2+^ interference therapy, a continuous supply of Ca^2+^ from the tumor microenvironment (TME) is essential. To this end, photothermal nanotransducers loaded with calcium‐based nanomaterials have been designed that can be activated by mild acidity and photothermal heating simultaneously. For instance, Ma et al. developed an intracellular Ca^2+^ cascade by constructing biodegradable PEGylated CaCO_3_ NPs as smart nanocarriers to deliver CuS nanodots to the tumor region via the enhanced permeability and retention (EPR) effect.^[^ ^39^ ^]^ CaCO_3_ nanocarriers gradually decomposed in the acidic TME and acted as a reservoir to supply abundant Ca^2+^. The released CuS nanodots exhibited NIR‐II (1064 nm) photothermal effect and stimulated TRPV1 for continuous Ca^2+^ influx in cancer cells (Figure 1B).

Given the low attenuation and negligible magnetic susceptibility of magnetic fields (0.1–1.0 MHz) in biological tissues, magnetothermal heating has been considered a promising complementary technology for temperature‐induced [Ca^2+^]_i_ regulation.^[^ ^40^, ^41^ ^]^ Conventionally, two‐component systems are required to stimulate cells with low TRPV1 expression: 1) viral infection for the introduction of genetically encoded channels; 2) the presence of nanoheaters. To simplify the systems, Stanley et al. devised a single‐component system by merging magnetothermal nanotransducers with TRPV1 channels.^[^ ^42^ ^]^ They designed three genetically encoded endogenous paramagnetic iron‐loaded ferritin NPs with varied proximity to TRPV1 channels: cytosolic ferritin, membrane‐associated ferritin, and TRPV1‐tethered ferritin (Figure 1C). The TRPV1‐tethered ferritin proved to be the most effective nanotransducer to elicit Ca^2+^ dynamics. Magnetothermal multiplexing has been recently identified to enable hierarchical regulation of Ca^2+^ dynamics in two cell or animal populations containing MNPs with distinct coercivities because each MNP could only respond to one certain AMF.^[^ ^43^, ^44^ ^]^ The Anikeeva group synthesized two MNPs responding to AMFs with different frequencies (*f*) and amplitudes (*H*) (Figure 1D). Fe_3_O_4_ NPs with low coercivity and high effective anisotropy responded to an AMF with high *f* but low *H* values (*H*:10 kA m^−1^, *f*: 522 kHz), whereas Co_0.24_Fe_2.76_O_4_ NPs with high coercivity and low effective anisotropy responded to an AMF with low *f* but high *H* values (70 kA m^−1^, 50 kHz).^[^ ^44^ ^]^ TRPV1‐expressing HEK293FT cells were submerged with different ferrofluids of MNPs and displayed AMF‐specific Ca^2+^ response. This strategy, however, has not been employed to modulate mixed cell culture simultaneously in this study. Notably, compared to photothermal heating, the temporal resolution of the magnetothermal process is comparatively slow.^[^ ^45^ ^]^ Replacing temperature‐sensitive Ca^2+^ channels with rate‐sensitive thermoreceptors may be a potential alternative.

In addition to activating TRPV1, local photoheating on the cell membrane can directly raise membrane capacitance, which elicits an inward depolarizing capacitive current, resulting in Ca^2+^ influx through voltage‐gated Ca^2+^ channels (VGCCs).^[^ ^10^ ^]^ Several photothermal nanotransducers such as black phosphorus nanosheets,^[^ ^46^ ^]^ nanowire‐templated 3D fuzzy graphene,^[^ ^47^ ^]^ silicon nanowires,^[^ ^48^ ^]^ and Au NPs^[^ ^49^ ^]^ have been employed for Ca^2+^ influx via this mechanism. For instance, Rastogi et al. fabricated a hybrid nanotransducer from 3D fuzzy graphene that was grown on an intrinsic Si nanowire template (referred to as NT‐3DFG).^[^ ^47^ ^]^ Intriguingly, NT‐3DFG exhibited around 95% absorption over the whole wavelength range (250–800 nm), necessitating one to two orders of magnitude lower laser energy than previously reported photothermal nanomaterials. In the 2D cell culture, NT‐3DFG adhered to the plasma membrane of primary rat dorsal root ganglion neurons (DRG) rather than being endocytosed. Illuminating the cell‐NT‐3DFG interface with laser pulses led to membrane depolarization, which caused action potential firing and Ca^2+^ influx in both 2D cell cultures and 3D cortical neural spheroids.

#### 2.1.2 Heating‐Activated Endogenous Ca^2+^ Release

Nanomaterials can be endocytosed by diverse cell types, allowing them to interact with organelles. Therefore, nanotransducers manipulate [Ca^2+^]_i_ by inducing Ca^2+^ release from some organelles including the endoplasmic reticulum (ER) and lysosome is also a feasible strategy. In recent years, light‐driven nanotransducers including polymer capsules,^[^ ^50^ ^]^ silicon nanowires,^[^ ^51^ ^]^ and liquid metal NPs, et al. have been observed to elicit Ca^2+^ dynamics. Among them, we for the first time reported [Ca^2+^]_i_ manipulation via lysosomal Ca^2+^ release‐induced Ca^2+^ release from ER.^[^ ^50^ ^]^ Polymer capsules integrated with plasmonic star‐shaped AuNPs were internalized to cells and resided in lysosomes. Upon laser irradiation, the lysosomal membrane was transiently ruptured to locally release a very small amount of Ca^2+^, which was sufficient to activate inositol 1,4,5‐trisphosphate receptors (IP_3_R) in nearby ER and further triggered a Ca^2+^ release cascade (Figure 1E). Stimulation on a single cell could result in robust intercellular Ca^2+^ waves in both 2D and 3D cell cultures, either via an ATP secretion or gap junction‐related process. In addition to photothermal heating alone, a single nanotransducer can produce mixed stimuli. The Miyako group prepared liquid metals NPs from gallium‐indium eutectic alloys and coated them with a lipid layer consisting of 1,2‐distearoyl‐sn‐glycero‐3‐phosphoethanolamine‐N‐[amino(polyethylene glycol)‐2000] and 1,2‐bis(10,12‐tricosadiynoyl)‐sn‐glycero‐3‐phosphocholine.^[^ ^52^ ^]^ After cellular uptake, liquid metal NPs exhibited the synergy effect of photothermal heating and reactive oxygen species (ROS) generation by laser irradiation, which rapidly burst [Ca^2+^]_i_ in both irradiated TRPV1‐expressing ND7/23 hybrid cells and TRVP1‐negative HeLa cells, as well as adjacent ND7/23 hybrid cells (Figure 1F). Tian and colleagues assumed that photo‐illumination of silicon nanowires caused a localized and transient temperature increase within surrounding cytosol and organelles, which either generated ROS or transiently perforated ER and mitochondrial membrane.^[^ ^53^ ^]^ Nonetheless, the exact mechanisms underlying intracellular photothermal heating‐triggered Ca^2+^ transients, especially in TRPV1‐negative cells, remain unresolved. For this, the Ishiwata team proposed that local temperature change, or more specifically, the recooling process after photothermal heating caused decreased Ca^2+^ uptake by sarcoplasmic/endoplasmic reticulum Ca^2+^ ATPase (SERCA) pump and increased Ca^2+^ release through IP_3_Rs simultaneously.^[^ ^54^, ^55^ ^]^

### 2.2 Photostimulation

**Figure 2:**
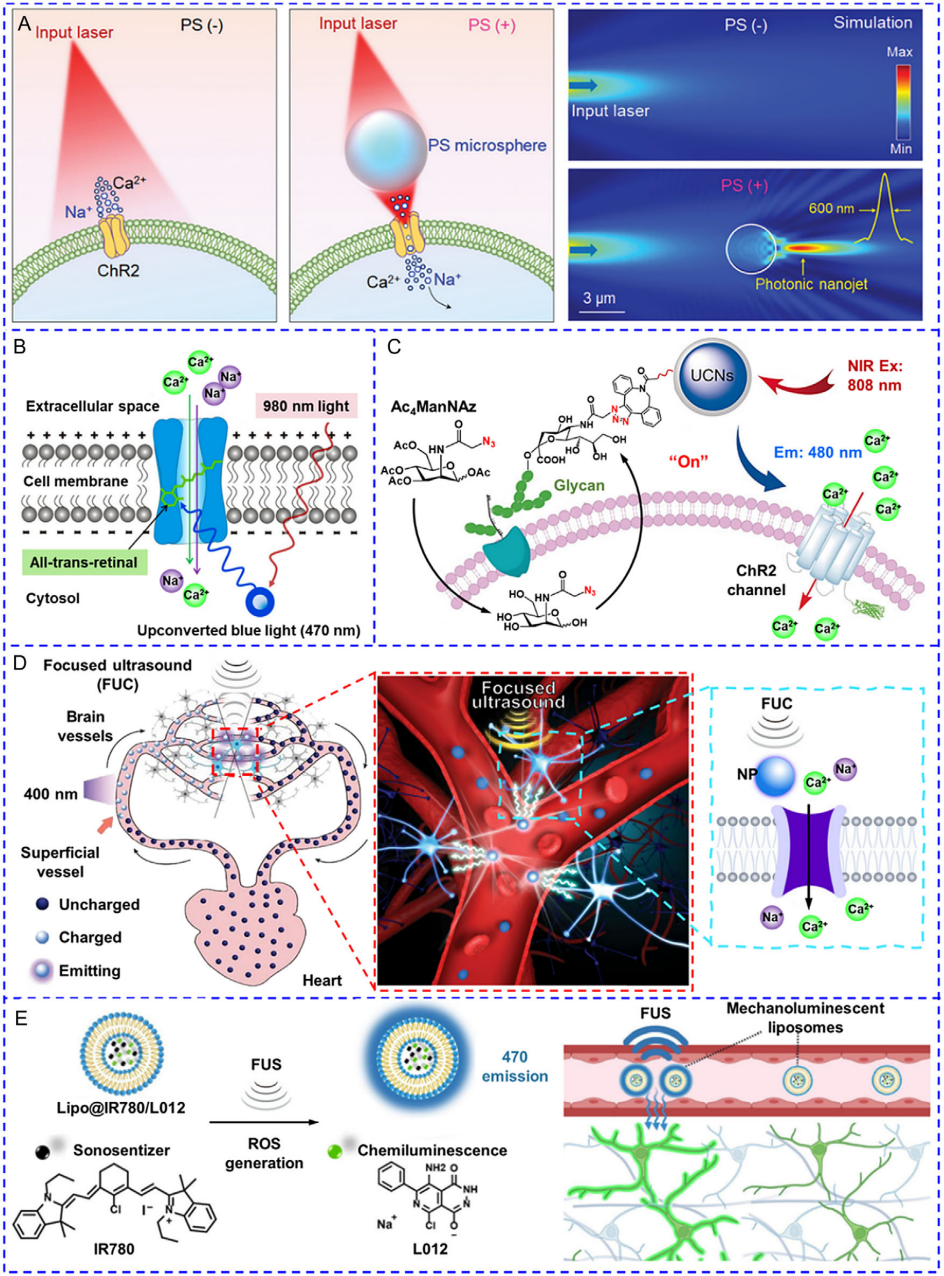
A) Schematic diagram of polystyrene microspheres as an optical lens to focus incident light for reducing input light intensity. Reproduced with permission.^[^ ^58^ ^]^ Copyright 2022, Wiley. B) Illustration of endocytosed UCNPs‐mediated optical stimulation of ChR2. Reproduced with permission.^[^ ^60^ ^]^ Copyright 2017, American Chemical Society. C) Scheme description of UCNPs binding to glycan on the cell membrane. Reproduced with permission.^[^ ^65^ ^]^ Copyright 2017, Wiley. D) Schematic diagram of chargeable mechanoluminescent NPs circulating in the blood vessels for stimulating ChR2‐expressing neurons in the brain. Reproduced with permission.^[^ ^67^ ^]^ Copyright 2019, United States National Academy of Sciences. E) Schematic diagram showing a cascade reaction inside mechanoluminescent liposomes for emitting blue light and activating ChR2‐expressing motor cortex neurons in mice. Reproduced with permission.^[^ ^68^ ^]^ Copyright 2023, American Chemical Society.

Although photothermal strategies have proven to modulate Ca^2+^ dynamics effectively, milder stimulation without damaging cells is still highly demanding. Optogenetics employing visible light‐gated cation channels is one of the groundbreaking innovations in the past decades.^[^ ^56^ ^]^ Light‐gated cation channels are transmembrane protein complexes capable of spatiotemporal regulation of cellular activities through optical control of ion mobilization across the cell membrane. They have been recognized as valuable tools for investigating neural communications and facilitating the development of therapeutic agents for disease treatment. Channelrhodopsin‐2 (ChR2), a non‐selective cation channel isolated from green algae, is permeable to Ca^2+^ and Na^+^ ions.^[^ ^57^ ^]^ Due to the conformational change of retinal between all‐trans and 13‐cis‐retinal, ChR2 responds swiftly and reversibly to blue light with a maximal response peak at ≈460 nm within milliseconds and a saturated laser density of ≈0.31 W cm^−2^ at 488 nm.^[^ ^58^ ^]^ To decrease the input laser energy and avoid the visible light‐induced heating effect, Guo et al. developed a micro‐optical lens based on polystyrene spheres (≈3 μm), which could effectively focus the incident light for increasing output light power on ChR2 channels in targeted cells.^[^ ^58^ ^]^ **Figure** **2**A displays that the power density of input light was significantly concentrated through polystyrene microspheres. Compared to cells without micro‐optical lenses, polystyrene microspheres greatly reduced the required power density threshold to induce action potentials and boosted the inward currents of target cells by nearly 132%.

However, visible light activation raises concerns regarding limited tissue penetration, as well as inherent absorption and scattering caused by endogenous chromophores including hemoglobin, flavins, and melanin.^[^ ^59^ ^]^ UCNPs can convert NIR light to UV or visible light and have been extensively used to activate light‐gated Ca^2+^ channels with deep tissue penetration and high stimulation efficiency.^[^ ^60^, ^61^, ^62^, ^63^, ^64^ ^]^ To achieve highly effective NIR‐to‐visible light conversion, Pliss et al. prepared a UCNP by coating an epitaxial shell layer of NaYF_4_ onto the surface of the NaYbF_4_:Tm core.^[^ ^60^ ^]^ UCNPs were further modified with folic acids as the targeting ligand to promote their internalization. As shown in Figure 2B, under 980 nm NIR irradiation, the upconverted blue light activated the ChR2 to facilitate the inflow of Ca^2+^ and Na^+^ ions. It is expected that randomly distributed UCNPs that are far from ChR2 channels will require a larger concentration of UCNPs or higher light intensity to deliver sufficient energy for ChR2 activation. Therefore, a strategy involving site‐specific localization, i.e., UCNPs in close proximity or directly binding to ChR2, is anticipated to enhance the manipulation efficiency and reduce thermal damage. The Xing group anchored UCNPs to the glycan on the cell surface through metabolic glycan‐biosynthesis pathways.^[^ ^65^ ^]^ As demonstrated in Figure 2C, a monosaccharide precursor (peracetylated N‐azidoacetylmannosamine) functionalized with an azido tag was introduced into glycoconjugates on the cell membrane, thereby providing a binding site for UCNPs with a modified dibenzyl cyclooctyne (DBCO) moiety via copper‐free click cyclization. NIR light (808 nm) enabled ChR2‐gated Ca^2+^ influx, which promoted Ca^2+^‐dependent apoptosis of HEK293 cells in 2D culture and zebrafish. Note that, conventional UCNPs still suffer from the extremely low upconversion quantum efficiency (≈0.2%), i.e., only ≈0.2% of the absorbed photons are transformed into visible emission.^[^ ^66^ ^]^ Consequently, high NIR light intensity is still required to achieve efficient photostimulation, which will inevitably raise the risk of nonspecific hyperthermia.

Mechanoluminescent NPs have also been designed to serve as local light sources in the brain that were triggered by brain‐penetrating focused ultrasound.^[^ ^67^ ^]^ After i.v. injection of mechanoluminescent NPs, a 400 nm excitation light source was put adjacent to the superficial artery and jugular vein to charge the circulating NPs when they passed through the illuminated region. Because of the interconnected network of vasculature and their accessibility to nearly all regions in the brain, NPs flowed past the focused ultrasound in the specific brain region and discharged their stored energy by emitting 470 nm light. The upconverted emission light stimulated ChR2‐expressing neurons that were in the vicinity of blood vessels (Figure 2D). The primary advantage of this work is that it does not need to cross the blood‐brain barrier, and it allows for repetitive stimulation due to the rechargeability of NPs. Recently, the same group introduced a cascade reaction into a liposome NP for ultrasound‐triggered mechanoluminescence in brain photon delivery.^[^ ^68^ ^]^ Sonosensitizer IR780 could generate ROS in the presence of acoustic cavitation, which then activated chemiluminescent L012 to emit blue light. Following intravenous administration of lipid nanoparticles, ultrasound irradiation permitted reversible activation of ChR2‐expressing motor cortex neurons in mice, resulting in limb movements.

### 2.3 Mechanical Stimulation

Mechanical force generated by focused ultrasound or magnetic field has been utilized to induce membrane depolarization or directly activate mechanosensitive cation channels, including Piezo‐type mechanosensitive ion channel component (Piezo), transient receptor potential canonical (TRPC), transient receptor potential melastatin 7 (TRPM7), transient receptor potential cation channel subfamily V member 4 (TRPV4), N‐type mechanosensitive Ca^2+^ channel, and mechanosensitive channel of small conductance (MscS) within the range of pN to nN.^[^ ^69^ ^]^ Two gating models, namely the lipid bilayer stretch model and the spring‐like tether model, have been proposed to illustrate the working principle of mechano‐sensitive channels.^[^ ^70^ ^]^ Mechanical stress (≈100 μN cm^−2^) is also reported to stimulate cells to release adenosine triphosphate (ATP) into extracellular space through ATP‐permeable hemichannels.^[^ ^71^, ^72^ ^]^ Following this, extracellular ATP (eATP) triggers Ca^2+^ influx by activating purinergic receptors on the cell membrane.^[^ ^73^, ^74^ ^]^ Nanotransducers with spatiotemporal controllability to activate mechanosensitive cation ion channels in complex biological systems have gained tremendous attention in the study of mechanotransductions involved in somatic sensation (e.g., touch and pain, pressure, balance, proprioception) and therapeutics such as mechano‐immunoengineering.^[^ ^75^, ^76^ ^]^ Thus, nanotransducers responding to magnetic fields, acoustic waves, and lights have been designed to mechanically manipulate [Ca^2+^]_i_.

#### 2.3.1 Magnetomechanical Activation of Mechanosensitive Cation Channels

**Figure 3:**
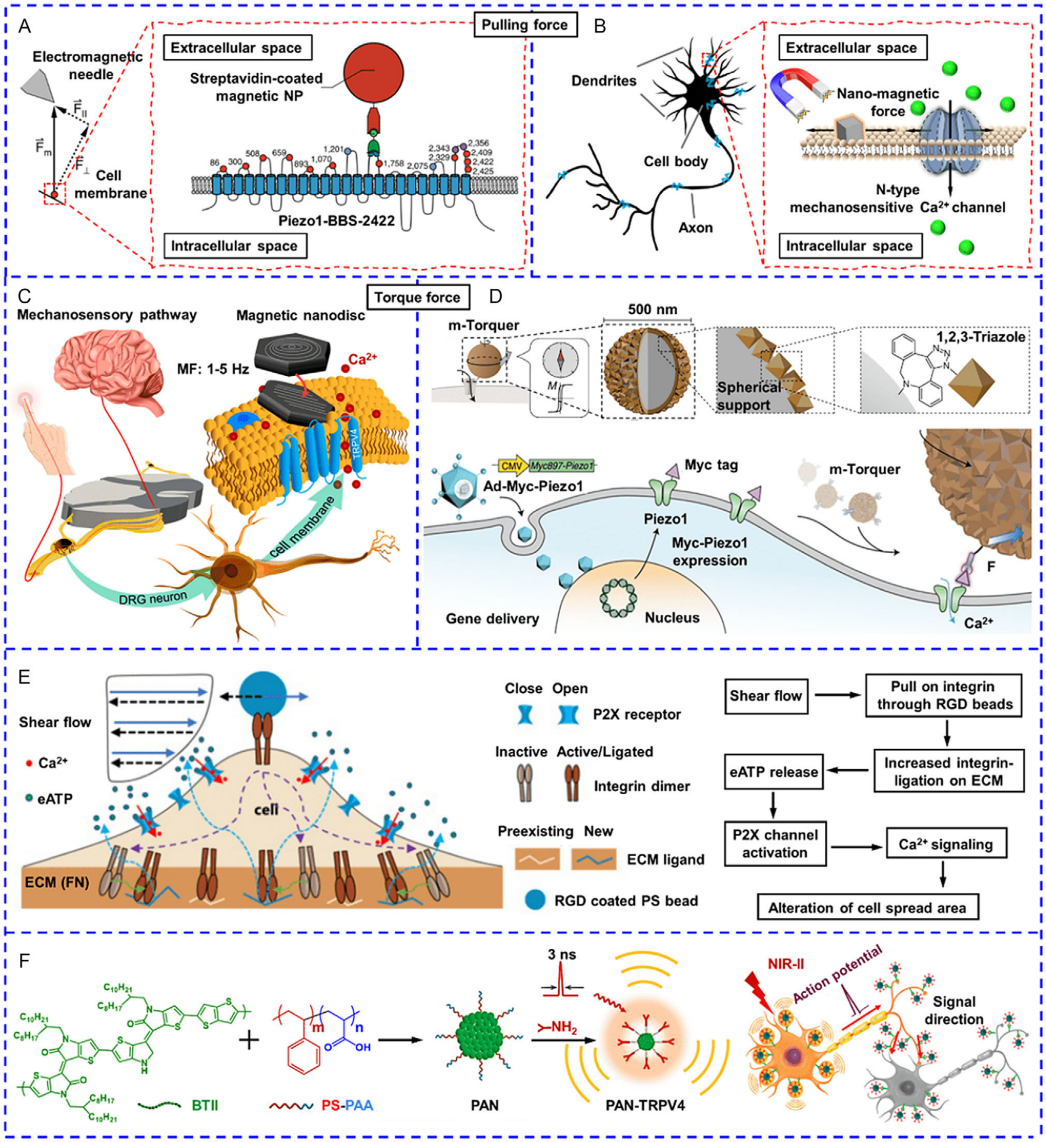
A) Schematic diagram of pulling forces produced by attaching MNPs to extracellular domains of Piezo1 for activating Piezo1. Reproduced with permission.^[^ ^79^ ^]^ Copyright 2016, Springer Nature. B) Working principle of membrane‐associated MNPs to open the N‐type mechanosensitive Ca^2+^ channels via lipid bilayer stretching. Reproduced with permission.^[^ ^81^ ^]^ Copyright 2016, American Chemical Society. C) Scheme of nanodiscs generating torque forces for activating TRPV4 and Piezo2 in dorsal root ganglion neurons. Reproduced with permission.^[^ ^85^ ^]^ Copyright 2020, American Chemical Society. D) Working flow of Piezo1‐targeting *m*‐Torquer system mediated channel opening for Ca^2+^ influx. Reproduced with permission.^[^ ^86^ ^]^ Copyright 2021, Springer Nature. E) The theoretically working mechanisms of using arginyl‐glycyl‐aspartic acid (RGD)‐modified PS nanobeads to enhance the non‐injury Ca^2+^ response. Reproduced with permission.^[^ ^92^ ^]^ Copyright 2021, Ivyspring International Publisher. F) The synthesis of photoacoustic nanotransducers (PANs) and their application to stimulate TRPV4 under the control of NIR irradiation for manipulating [Ca^2+^]_i_. Reproduced with permission.^[^ ^97^ ^]^ Copyright 2021, Cell Press.

Generally, the activation of mechanosensitive cation channels can be categorized into two types. The first type is to convert external magnetic fields into local pulling or torque forces. When a magnet is placed near MNPs, they will move toward the source of the magnetic field to produce pulling forces that can activate mechanosensitive cation channels and cause Ca^2+^ influx.^[^ ^77^, ^78^, ^79^, ^80^, ^81^, ^82^ ^]^ Grandl's group attached MNPs directly to distinct extracellular domains of the Piezo1 channel and applied localized pulling forces through the electromagnet (**Figure** **3**A).^[^ ^79^ ^]^ They discovered that four extracellular sites within two domains were mechanically sensitive. In addition to having a direct influence on mechanosensitive channels, pulling forces acting on other regions of the membrane can also transduce stretching forces to mechanosensitive channels.^[^ ^80^, ^81^, ^83^ ^]^ Tay et al. exploited starch‐coated MNPs that were primarily associated with the cell membrane to stimulate primary cortical neurons on a magnetic chip with a magnetic field gradient.^[^ ^81^, ^83^ ^]^ Nanomagnetic force ranging from 0.1 to 1 nN was sufficient to open the N‐type mechanosensitive Ca^2+^ channels via lipid bilayer stretching, thereby inducing Ca^2+^ influx and the propagation of intracellular Ca^2+^ waves along neurites (Figure 3B). Long et al. genetically engineered cells to express exogenous magnetoreceptors, namely iron‐sulfur cluster assembly proteins, in HEK293 cells and hippocampal neurons.^[^ ^84^ ^]^ In the presence of a pair of bar magnets or electromagnetic coils, a constant magnetic field was generated. When magnetic strength exceeded 0.3 mT, the cell membrane depolarized, and Ca^2+^ entered the cytosol. To circumvent the difficulty of direct conversion of magnetic anisotropy energy to pulling force, the Cheon team utilized a two‐step strategy involving magnetic‐to‐thermal and thermal‐to‐mechanical transduction. The magnetic nanoparticle cluster core within the hydrogel nanoparticles generated local magnetothermal heating, induced the contraction of the surrounding thermo‐responsive poly‐N‐isopropylmethylacrylamide outer layer, and ultimately delivered forces to activate targeted mechanoreceptors.^[^ ^82^ ^]^ In most current studies, magnetic nanotransducers were directly bound to mechanosensitive channels or associated with the cell surface, and only a few studies revealed that internalized magnetic nanotransducers could also elicit Ca^2+^ responses.^[^ ^77^ ^]^

Recent reports described nanotransducers that generated torque forces to activate mechanosensitive cation channels.^[^ ^85^, ^86^, ^87^, ^88^, ^89^ ^]^ In a slowly rotating direct current (DC) field, magnetic nanotransducers will rotate to align with the field direction, thereby generating torque forces.^[^ ^12^ ^]^ Gregurec et al. synthesized magnetic nanodiscs to exert tunable torque forces on the cell surface in the presence of a weak (26 mT) and a slowly rotating magnetic field (5 Hz).^[^ ^85^ ^]^ The torque was produced through the transition from a vortex to an in‐plane magnetization state, which was proportional to the magnetic moment and the strength of the magnetic field. Nanoscale torque was adequate to activate several types of ion channels, including TRPV4 and Piezo2, facilitating remote control of Ca^2+^ influx in DRG neurons, whereas magnetic nanodiscs failed to activate non‐mechanosensitive HEK293 cells (Figure 3C). Cheon's group introduced a Piezo1‐targeting magnetic torquer (*m*‐Torquer) system for non‐contact long‐range magnetic stimulation of Piezo1 both in vitro and in vivo.^[^ ^86^ ^]^ The *m*‐Torquer was assembled by decorating octahedral MNPs onto a spherical polystyrene substrate via copper‐free click reaction (Figure 3D). In the presence of the circular magnet array that generated a rotating weak uniform magnetic field (0.5 Hz), *m*‐Torquer produced torque forces of 1.6 pN and triggered Piezo1‐dependent Ca^2+^ influx in mouse primary cortical neurons. Pulsed application of circular magnet array could even achieve repetitive activation of Piezo1 and consecutive Ca^2+^ spiking. To simplify the multicomponent systems, Wheeler et al. developed Magneto 2.0, a single‐component system that integrated TRPV4 channels with iron‐containing ferritin NPs and a plasma membrane trafficking signal.^[^ ^90^ ^]^ They assumed that the magnetic torque of the tethered ferritin activated TRPV4 channels in the presence of a magnetic field (50 mT) elevated the cytosolic Ca^2+^ level.

#### 2.3.2 Activation by Ultrasound and Cavitation Effect

High‐frequency ultrasound (30–150 MHz) can directly activate the Piezo1 channels, but its in vivo application is severely hampered by the short working distance (<5 mm).^[^ ^91^ ^]^ In contrast, low‐frequency ultrasound (1–2 MHz) has a longer working distance (a few centimeters) but is inadequate to activate Piezo1.^[^ ^92^ ^]^ Furthermore, the diffraction‐limited spatial resolution of conventional ultrasound modulation is in the millimeter to centimeter range.^[^ ^93^ ^]^ Therefore, the second type of mechanical stimulation approach is to introduce ultrasound‐responsive nanotransducers as the localized force actuators that can dramatically enhance the ultrasonic effect as well as the precision and controllability of low‐frequency ultrasound‐induced [Ca^2+^]_i_ manipulation. For example, the Sun group isolated gas vesicles with hollow protein shells from *Anabaena ﬂos‐aquae* as a nanosized ultrasound contrast agent, which could induce Ca^2+^ influx and neuronal activation in the targeted neurons both in vitro and in vivo.^[^ ^94^, ^95^ ^]^ Low‐intensity ultrasound‐induced buckling effects transmit mechanical perturbations to mechanosensitive cation channels in primary cortical neurons, with dose‐dependent and reversible neuronal responses.

Apart from ultrasound, laser irradiation on specific nanotransducers can also cause a cavitation effect. Zhong and coworkers produced tandem microbubbles in situ to induce jetting flow, which caused the slight displacement of the integrin‐targeting microbeads and local membrane stretch.^[^ ^96^ ^]^ The mechanical stress was then transmitted through the cell surface to activate TRPM7 ion channels, initiating the intracellular Ca^2+^ wave that propagated from the endoplasmic reticulum to the cytosol via a reaction‐diffusion process. Interestingly, the same group used a similar experiment set‐up in 2021 but proposed a different mechanism.^[^ ^92^ ^]^ A single cavitation microbubble was used to stimulate integrin‐targeting microbeads on Piezo1‐negative HEK293T cells. The movement of microbeads induced shear force on integrins on the cell apical surface, leading to increased integrin ligation to the substrate extracellular matrix and consequent release of extracellular ATP. Eventually, ATP‐ activated P2X receptors and induced Ca^2+^ influx (Figure 3E). However, it remains challenging to apply this technique in vivo due to the rapid clearance of microbeads. Photoacoustic nanomaterials, including AuNPs and semiconducting polymers, have been fabricated to convert picosecond or nanosecond pulsed light into acoustic waves. Jiang et al. incorporated NIR‐II absorbing semiconducting polymer within polystyrene‐block‐poly(acryl acid) (PS‐b‐PAA) NPs to stimulate rat primary cortical neurons.^[^ ^97^ ^]^ Upon excitation by a 3‐ns pulsed laser, the TRPV4‐targeting NPs anchored on the neural soma and neurite effectively yielded a localized acoustic wave with a peak‐to‐peak pressure of 58.2 Pa at 10 nm from the NPs surface, eliciting action potentials and transient Ca^2+^ response (Figure 3F). Notably, continuous‐wave laser irradiation of NPs only produced photothermal effects without photoacoustic signals, and both longer irradiation duration and higher laser power were required to achieve a similar stimulation effect, indicating photoacoustic stimulation was a more gentle and sensitive method than photothermal effect.

### 2.4 Electrical Stimulation

VGCCs are a class of highly Ca^2+^‐selective cation channels that couple electrical signals on the cell surface to cellular physiology, including muscle contraction, neuronal firing, and neurotransmitter release. In excitable cells, VGCCs are activated upon membrane depolarization, followed by Ca^2+^ influx and the generation of an action potential. The inflowing Ca^2+^ further activates the ryanodine receptor (RyR) in the endo/sarcoplasmic reticulum for Ca^2+^ release.^[^ ^98^ ^]^ Targeting VGCCs has therapeutic implications for treating cardiovascular disorders, neurological and psychiatric diseases, regenerative medicine, and cancer treatment.^[^ ^14^, ^99^ ^]^

**Figure 4:**
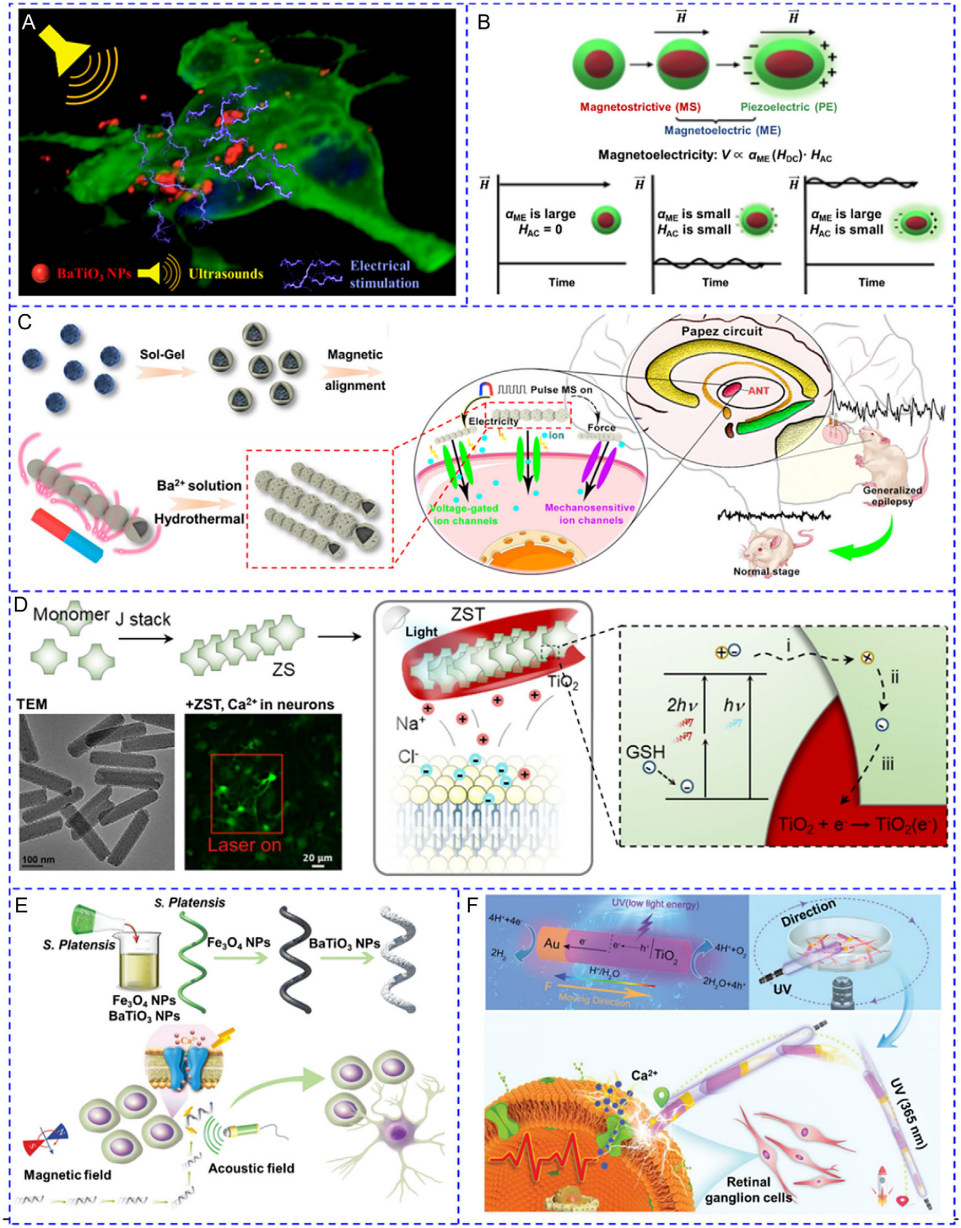
A) Ultrasound stimulated tetragonal barium titanate (tBaTiO_3_) NPs to generate electrical signals for inducing Ca^2+^ influx. Reproduced with permission.^[^ ^103^ ^]^ Copyright 2015, American Chemical Society. B) The schematic for the difference between using a large DC magnetic field and an AC magnetic field to generate an optimal magnetoelectric signal. Reproduced with permission.^[^ ^112^ ^]^ Copyright 2021, American Association for the Advancement of Science. C) Nanochains assembled from individual Fe_3_O_4_@BaTiO_3_ NPs produced higher electrical output to activate voltage‐gated and mechanosensitive ion channels in neurons. Reproduced with permission.^[^ ^113^ ^]^ Copyright 2023, American Chemical Society. D) Nanoscale optoelectrode based on TiO_2_ coated zinc porphyrin nanorods generated non‐Faradaic current to cause membrane depolarization and the generation of action potential. Reproduced with permission.^[^ ^118^ ^]^ Copyright 2024, Springer Nature. E) The flow of the preparation of *S.platensis*@Fe_3_O_4_@tBaTiO_3_ micromotor and the working principles of this micromotor in response to an acoustic signal to trigger Ca^2+^ response. Reproduced with permission.^[^ ^109^ ^]^ Copyright 2020, Wiley. F) TiO_2_‐Au nanowires were prepared to perform a photoelectric effect to manipulate [Ca^2+^]_i_ of neuronal retinal ganglion cells. Reproduced with permission.^[^ ^121^ ^]^ Copyright 2021, Wiley.

Current electrical implants, including invasive electrodes and devices, differ structurally and mechanically from their neuron targets, and the resulting tissue disruption prevents their stable operation and long‐term neuronal regulation.^[^ ^40^ ^]^ To reduce the invasiveness, a series of electrodeless nanogenerators that transform external input energy including ultrasound, magnetic field, or light, into electrical stimulation have been developed. Particularly, piezoelectric nanotransducers are very promising tools for wireless, noninvasive, and localized Ca^2+^ activation. In the physiological environment, transient deformations or structural anisotropy of piezoelectric NPs triggers charge separation, which generates local and transient electric fields close to the interface between NPs and electrolytes.^[^ ^100^ ^]^ Ultrasound waves are the most commonly used external stimulus to mechanically activate piezoelectric NPs, even though the underlying mechanism is not fully understood.^[^ ^14^ ^]^ Both inorganic and organic piezoelectric nanotransducers have been widely used for [Ca^2+^]_i_ regulation, including BaTiO_3_ NPs,^[^ ^101^, ^102^, ^103^, ^104^, ^105^ ^]^ boron nitride nanotubes,^[^ ^106^ ^]^ nylon‐11 NPs,^[^ ^107^ ^]^ and MoS_2_ nanosheets.^[^ ^108^ ^]^ In vitro, it has been reported that both intracellular and extracellular piezoelectric nanotransducers could regulate Ca^2+^ response in diverse cell types, such as neuron‐like PC‐12 cells, neuroblastoma‐derived SH‐SY5Y cells, myoblast, cancer cells, stem cells, and vascular endothelia.^[^ ^104^, ^105^, ^107^, ^109^, ^110^, ^111^ ^]^ As an example, Ciofani's group synthesized membrane‐associated tetragonal BaTiO_3_ NPs for investigating ultrasound‐triggered electrical stimulation of SH‐SY5Y cells. Ultrasonic stimulation of BaTiO_3_ NPs caused high‐amplitude Ca^2+^ transients.^[^ ^103^ ^]^ Both voltage‐gated Ca^2+^ and Na^+^ channels were indispensable for Ca^2+^ influx, as proved by pharmaceutical blockers (**Figure** **4**A).

Magnetoelectric nanotransducers integrate piezoelectric and magnetostrictive materials and efficiently transfer magnetic forces into local electric fields.^[^ ^112^, ^113^, ^114^ ^]^ Magnetoelectric NPs can redistribute surface charges and produce electrical output in response to magnetic stimuli; this results in the depolarization of the plasma membrane and activation of nearby cells. Kozielski et al. developed two‐phase magnetoelectric nanotransducers by covering magnetostrictive CoFe_2_O_4_ NPs with piezoelectric BaTiO_3_ NPs^[^ ^112^ ^]^ (Figure 4B). The magnetic field created strain in CoFe_2_O_4_, which was transduced to BaTiO_3_ and generated a charge separation. As a result, ≈20.1% of SH‐SY5Y cells exhibited Ca^2+^ transient in response to simultaneous alternating current (AC) and direct current (DC) magnetic field, but the AC or DC magnetic field alone failed to produce an adequate magnetoelectric effect. To enhance electrical output, the Fan team aligned individual Fe_3_O_4_@BaTiO_3_ NPs into nanochains, which dramatically increased coupling area and stress concentration between the magnetostrictive core and piezoelectric shell^[^ ^113^ ^]^ (Figure 4C). For the treatment of epilepsy, the nanochains were formed by a facile static‐magnet‐field assisted interface coassembly method. These nanochains modulated neuronal activity by synergistically activating voltage‐gated and mechanosensitive ion channels in the anterior nucleus of thalamus.

Nanoscale optoelectrodes, such as silicon nanowires,^[^ ^51^, ^115^ ^]^ carbon nanotubes,^[^ ^116^ ^]^ and quantum funnels,^[^ ^117^ ^]^ can directly transform light into electricity, enabling the manipulation of individual cells with subcellular precision. Based on the charge‐injection reactions, the current can be divided into faradaic and non‐faradaic (capacitive). To fabricate a non‐faradaic optoelectrode, the Bu group assembled zinc porphyrin into J‐aggregated nanorods and applied a TiO_2_ coating to the surface^[^ ^118^ ^]^ (Figure 4D). Nanorods allowed long‐range exciton diffusion, and the photoexcited electrons were rapidly transferred to TiO_2_. The formed TiO_2_(e^−^) attracted the cations around the neural membrane and decreased its potential, thereby causing membrane depolarization and the generation of action potential. The advantage of non‐faradaic optoelectrode is to avoid water hydrolysis and the formation of harsh hydroxyl radicals at the electrode–tissue interfaces, which is beneficial for long‐term neurostimulation.

Micromotors are revolutionary robotic systems enabling highly controllable navigation in the biological environment to precisely reach the target cells and can be driven by diverse energy sources. So far, only a few studies have explored their potential as mobile energy converters for [Ca^2+^]_i_ regulation.^[^ ^119^, ^120^ ^]^ Indeed, the combination of electrical nanotransducers and micromotors is expected to achieve single‐cell targeting motion and precise modulation of Ca^2+^ signaling.^[^ ^14^ ^]^ For instance, Liu et al. integrated BaTiO_3_ and Fe_3_O_4_ NPs onto a spiral‐shaped *S. platensis* template (Figure 4E).^[^ ^109^ ^]^ Under the guidance of a rotating magnetic field, micromotors moved toward a target PC‐12 cell at 333.3 μm s^−1^ and locally produced electrical signals in the presence of ultrasound. To further simplify the system by applying only one stimulus, Chen et al. recently developed a UV irradiation‐driven micromotor based on photoelectrochemical TiO_2_‐Au nanowires for precise control of single‐neuron activity.^[^ ^121^ ^]^ As shown in Figure 4F, during light irradiation (365 nm, 50 mW cm^−2^), H^+^ flux with fluids flowed toward the Au side, and double the amount of gas was generated on the Au side compared to the TiO_2_ side, both of which were responsible for propelling the micromotors toward the TiO_2_ side. Motors could navigate following the predesigned paths by varying the orientation of light. After reaching the targeted neuronal retinal ganglion cells, the potential difference of the entire motor (1.75 V) was sufficient to activate the VGCCs and elevate [Ca^2+^]_i_.

### 2.5 Chemical Stimulation

A variety of small molecule compounds, including capsaicin, allyl isothiocyanate (AITC), cannabis‐based terpenes, IP_3_, nicotinic acid adenine dinucleotide phosphate (NAADP), ATP, dopamine, SKF‐81 297, ROS, NO, curcumin, and kaempferol‐3‐O‐rutinoside, have been reported to target specific Ca^2+^ channels for regulating [Ca^2+^]_i_ (**Table** **1**). The direct use of these chemical modulators to stimulate Ca^2+^ channels both in vitro and in vivo is associated with low activation efficiency, severe toxicity, a lack of spatiotemporal control, and the requirement for multiple administrations.^[^ ^122^ ^]^ The introduction of nanotechnology was originally proposed to alter the pharmacokinetics and toxicity profiles of chemical modulators. The ability to concentrate chemical modulators within target cells and their corresponding Ca^2+^ channels is crucial for enhancing stimulation efficiency. Moreover, emerging nanomaterials have brought novel activation mechanisms for [Ca^2+^]_i_ regulation, including the local release of Ca^2+^, the in situ liberating of chemical modulators in response to environmental or external stimuli, and the catalytic production of chemical modulators. Owing to the tremendous advance in nanomaterials‐mediated chemical stimulation, we attempt to comprehensively cover this area, from their basic fabrication rationale to working principles in this section. We highlight the underlying mechanisms of how to use nanomaterials to improve the efficiency of manipulating [Ca^2+^]_i_ through representative samples.

#### 2.5.1 Nanoplatform‐Mediated Gradual Release of Pre‐Loaded Chemical Stimuli

Given the poor water solubility or cell impermeability of most chemical modulators, preloading them within nanomaterials, such as porous nanomaterials, polymer NPs, and liposomes, greatly increases their stability and bioavailability, and permits sustained and controlled release at target sites.^[^ ^123^, ^124^ ^]^ Francisco's team used poly(ethylene glycol)‐poly(lactic*‐co*‐glycolic acid) (PEG‐PLGA) NPs to encapsulate three cannabis‐based terpenes (i.e., β‐caryophyllene, β‐myrcene, and nerolidol).^[^ ^125^ ^]^ Terpenes delivered by polymer NPs activated TRPV1 more efficiently and resulted in a more robust rise in cytosolic Ca^2+^ level than free terpenes. However, this strategy is gradually fading out of the stage in recent years owing to the potential of off‐target effects posed by uncontrolled release in vivo application, and the emergence of nanotechnologies that enable more advanced and sophisticated designs.

#### 2.5.2 Controlled Release in Response to Microenvironmental Stimuli

**Figure 5:**
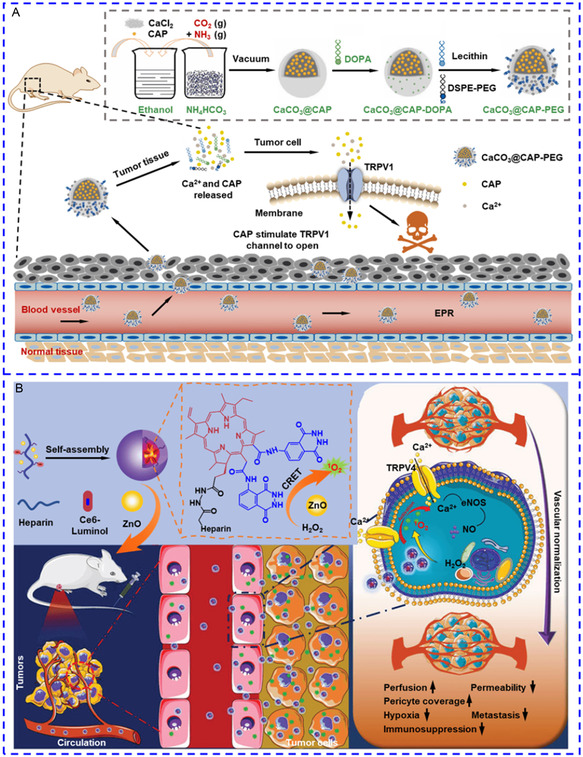
A) The preparation process of CaCO_3_@CAP‐PEG NPs and the working principles of the nanotransducers for cancer therapy. Reproduced with permission.^[^ ^133^ ^]^ Copyright 2022, Elsevier. B) Low molecular weight heparin‐Ce6‐luminol and ZnO NPs were integrated to synthesize a ^1^O_2_ generator for activating TRPV4‐gated Ca^2+^ influx. Reproduced with permission.^[^ ^134^ ^]^ Copyright 2022, Wiley.

Recently, tissue‐ or particularly TME‐responsive nanomaterials have been developed to meet the ever‐growing demand for precision medicine. These nanomaterials disintegrate in response to TME stimuli, such as pH, and H_2_O_2_, releasing their components and preloaded reagents.^[^ ^126^ ^]^ Consequently, these nanomaterials have been introduced to improve the efficiency of regulating Ca^2+^ signaling attributed to their capability of precise and controlled manipulation. Calcium‐based nanomaterials featuring superior biocompatibility and sensitive acidity responsiveness have drawn remarkable interest in drug delivery.^[^ ^19^ ^]^ The intrinsic calcium components further enhance the attractiveness for [Ca^2+^]_i_ regulation. To date, diverse calcium‐based nanomaterials, including CaF_2_,^[^ ^127^ ^]^ CaCO_3_,^[^ ^128^ ^]^ CaO_2_,^[^ ^129^ ^]^ Ca_3_(PO_4_)_2_,^[^ ^130^ ^]^ and Ca_2_SiO_4_^[^ ^131^ ^]^ NPs, have been exploited as exogenous Ca^2+^ suppliers. These NPs can be taken up directly by cells and trafficked to the highly acidic lysosomes (pH ≈ 4.5), where they are gradually dissolved and continually release Ca^2+^.^[^ ^132^ ^]^ The elevated osmotic pressure ruptures the lysosomal membrane, thereby releasing Ca^2+^ into the cytosol. Except for direct intracellular Ca^2+^ release, calcium‐based nanomaterials can also be degraded outside cells and deliver chemical agonists for Ca^2+^ influx. Xu et al. developed capsaicin‐loaded CaCO_3_ nanotransducers for cancer therapy.^[^ ^133^ ^]^ CaCO_3_ NPs were partially degraded in extracellular acidic TME (pH ≈ 6.5), thereby providing adequate extracellular Ca^2+^ for enhanced capsaicin‐mediated Ca^2+^ influx via TRPV1 channels (**Figure** **5**A). These methods lead to several h of sustained Ca^2+^ release/influx and maintenance of high [Ca^2+^]_i_, which are especially ideal for Ca^2+^ overload‐based cancer treatment. More details are systematically described in Section 3.1.

Owing to the aberrant metabolic activity in malignant tumors, ROS levels within tumor tissue can reach up to 100 μM, nearly five times that in normal tissue, which are potential candidates for ROS‐responsive nanotransducers to trigger Ca^2+^ dynamics.^[^ ^134^, ^135^ ^]^ For example, Zhang et al. fabricated an H_2_O_2_‐triggered self‐illuminated photodynamic nanotransducer to activate TRPV4 channel.^[^ ^134^ ^]^ Prior to self‐assembling on the surface of ZnO nanoezymes, photosensitizer chlorin e6 was first reacted with luminol, and the resulting product was then conjugated to heparin to form an amphiphilic macromolecule. ZnO nanoenzymes catalyzed the luminol‐H_2_O_2_ reaction, and the chemiluminescent resonance energy transferring from luminol to chlorin e6 (Ce6) efficiently generated ^1^O_2_ to open TRPV4 on tumor‐derived endothelial cells (Figure 5B). In fact, sufficient production and accumulation of biological microenvironmental stimuli is a prerequisite for constructing precise and controllable nanotransducers that respond specifically in the regions of target cells. This undoubtedly restricts the application scope of this strategy, promoting diligent exploration of more versatile nanotransducers for precise and controlled release of chemical stimuli to regulate [Ca^2+^]_i_.

#### 2.5.3 Controlled Release in Response to the External Energy Field

**Figure 6:**
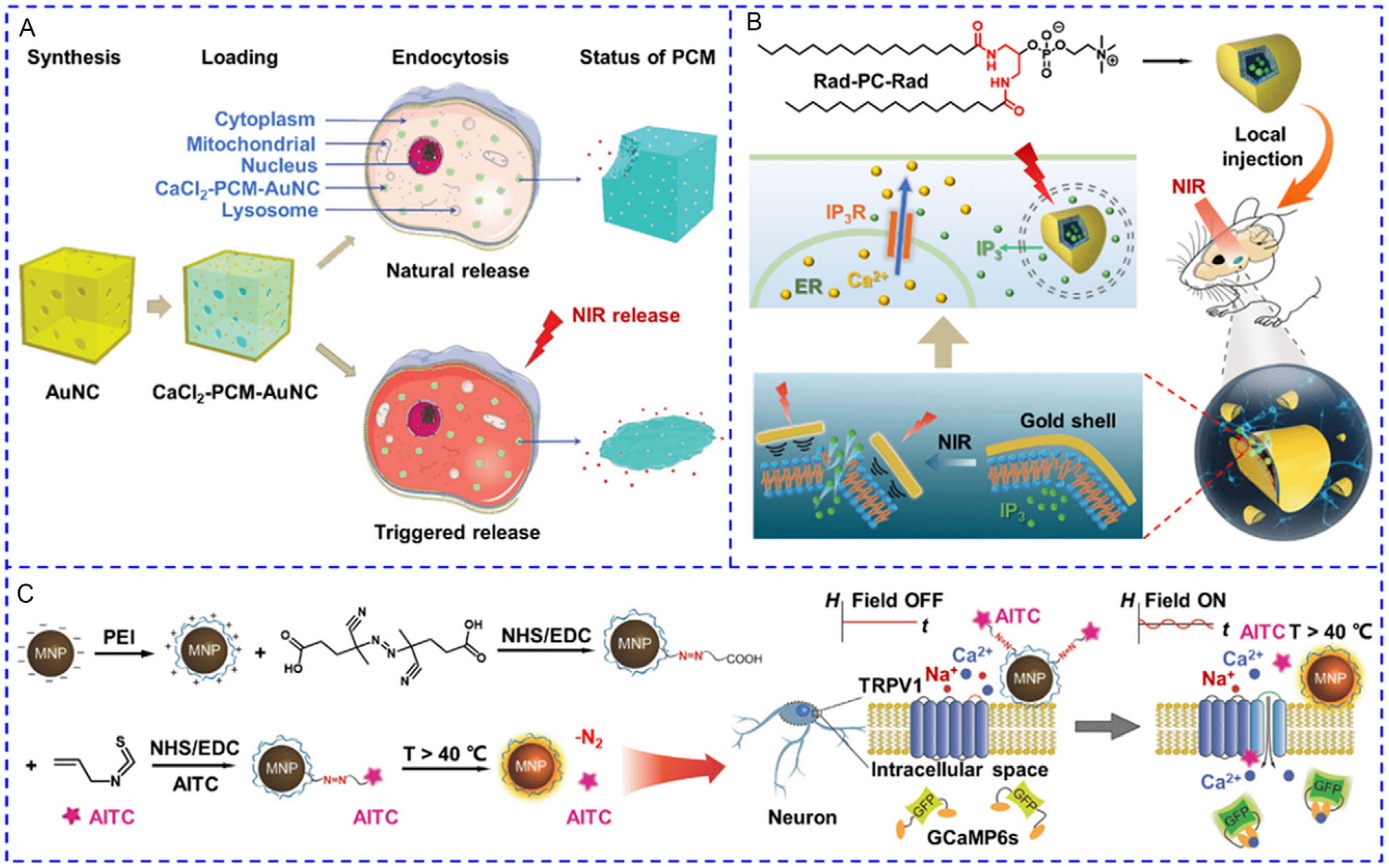
A) The Au nanocage (AuNC) was loaded with CaCl_2_ and the surface pores were sealed by phase change materials. Once entering cancer cells, the nanotransducers responded to NIR and generated a photothermal effect to release CaCl_2_ for Ca^2+^ overload. Reproduced with permission.^[^ ^136^ ^]^ Copyright 2019, Wiley. B) The gold shell of mechanoresponsive nanovesicles can be disintegrated under NIR irradiation to release IP_3_ to stimulate IP_3_R on the endoplasmic reticulum and induce a rise in [Ca^2+^]_i_. Reproduced with permission.^[^ ^140^ ^]^ Copyright 2020, Wiley. C) AITC was conjugated to magnetic beads via a heat‐sensitive link. The magnetothermal effect mediated by magnetic beads could release AITC to activate TRPV1 for Ca^2+^ influx. Reproduced with permission.^[^ ^141^ ^]^ Copyright 2016, Wiley.

Replacing microenvironmental stimuli with external energy fields to remotely trigger nanotransducers has broader applications because of the elimination of biological restrictions and the on‐demand release of chemical modulators. Nanotransducers can be categorized into three types according to their stimulation manners. The first type of nanotransducers unloads chemical modulators in a nonrepetitive “one‐shot” fashion. Ca^2+^ or chemical modulators can be physically encapsulated into hollow nanomaterials, including Au nanocage,^[^ ^136^ ^]^ mesoporous silica NPs,^[^ ^137^ ^]^ plasmonic liposomes,^[^ ^138^ ^]^ and plasmonic nanocapsules.^[^ ^139^ ^]^ For instance, the Xia group encapsulated calcium chloride (CaCl_2_) into Au nanocages, and the surface pores were sealed with lauric acid, a phase change material melting at 43 °C.^[^ ^136^ ^]^ After photothermal heating of the nanocage for 10 min, lauric acid melted and released Ca^2+^ disrupted cytosolic Ca^2+^ homeostasis (**Figure** **6**A). To boost the photosensitivity and reduce response time to seconds or even milliseconds, plasmonic liposomes are chosen for loading chemical modulators, as they only necessitate ps‐to‐ms laser irradiation. Xiong and colleagues designed an ultra‐photosensitive nanovesicle capable of delivering cargo to deep brain regions.^[^ ^140^ ^]^ The photo‐controlled release was achieved by decorating the synthetic phospholipid (Rad‐PC‐Rad) with a gold shell. Single ultrashort laser pulse irradiation (28 ps) induced nanomechanical stress to destroy the integrity of the nanovesicle and release IP_3_ for activating IP_3_R on ER (Figure 6B). Additionally, chemical stimuli can be chemically bonded to polymer or inorganic NPs via thermal^[^ ^141^ ^]^ and ROS‐cleavable linkage.^[^ ^142^ ^]^ Romero et al. fabricated a potent TRPV1 nano‐antagonist by coupling AITC, a TRPV1 agonist, to MNPs with a thermally labile azo‐linker.^[^ ^141^ ^]^ As can be seen in Figure 6C, exposure to an AMF swiftly released virtually the entire payload within 7 s, and the released AITC could activate TRPV1 in hippocampal neurons for Ca^2+^ influx.

**Figure 7:**
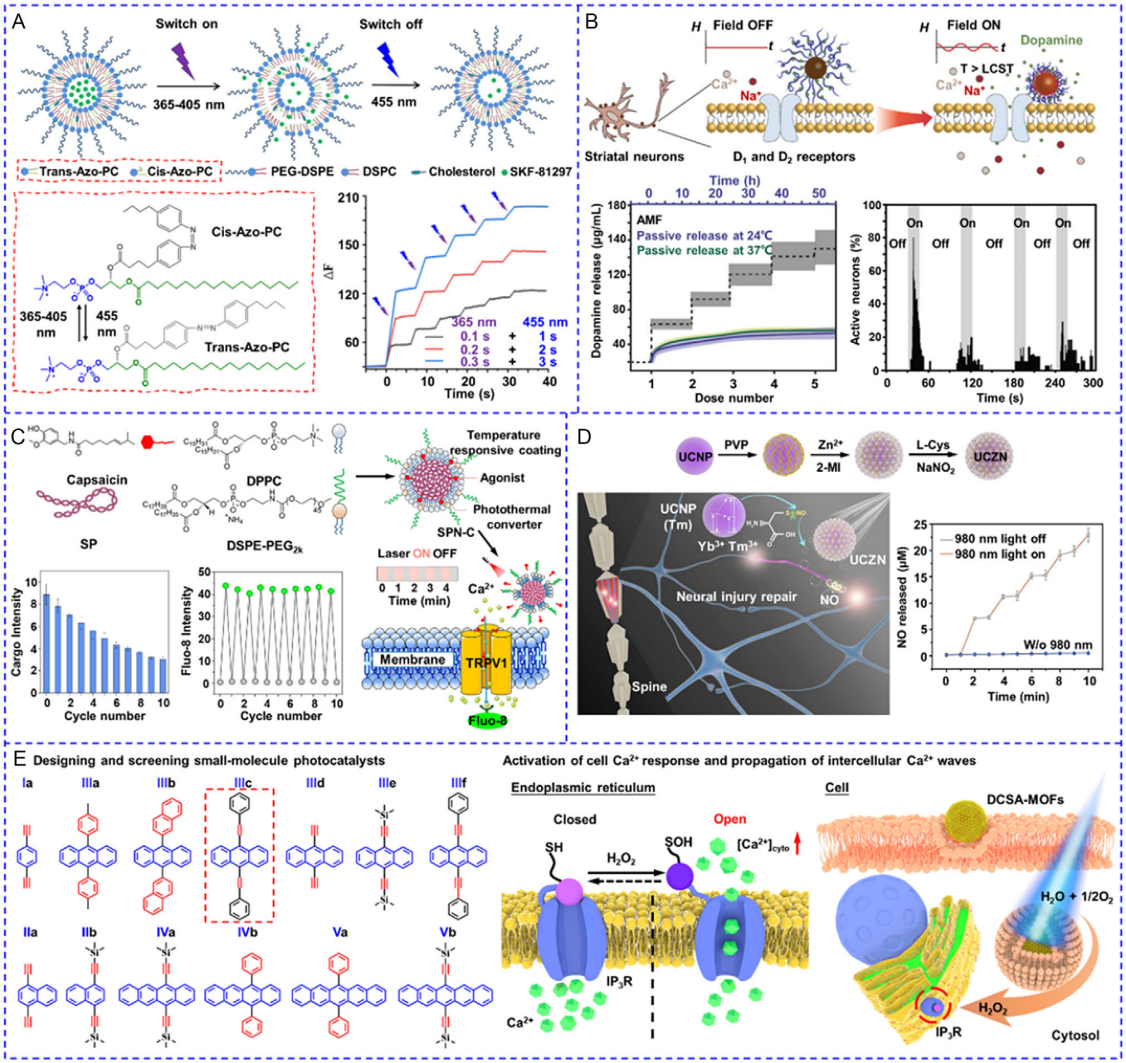
A) UV and blue light‐regulated the switch on and off of photoswitchable azobenzene‐containing liposome and the release of SKF‐81297 to activate D1 receptors. Reproduced with permission.^[^ ^143^ ^]^ Copyright 2022, Tsinghua University Press. B) Magnetothermal effect of MNPs raised the local temperature to the low critical solution temperature of the polymer and released pre‐loaded dopamine to activate D1 and D2 receptors. Reproduced with permission.^[^ ^144^ ^]^ Copyright 2022, Wiley. C) 808 nm irradiation could increase the local temperature to cause the phase transition of DPPC lipidic NPs and release the pre‐loaded capsaicin to stimulate TRPV1 channels. Reproduced with permission.^[^ ^146^ ^]^ Copyright 2018, American Chemical Society. D) NIR light‐mediated upconverted emission from UCNP to liberate NO from ZIF‐8 MOF and activate RyR receptors on the endoplasmic reticulum. Reproduced with permission.^[^ ^147^ ^]^ Copyright, American Association for the Advancement of Science. E) Photocatalytic activation of IP_3_R for regulating cellular and animal behaviors via MOF‐enabled H_2_O_2_ generation. Reproduced with permission.^[^ ^152^ ^]^ Copyright 2024, American Association for the Advancement of Science.

The second type of nanotransducers enables the switchable and repetitive release of chemical modulators by employing light/thermal reversible response materials. As an example, the Qin group produced azobenzene‐containing photoswitchable liposomes for controlling the release of SKF‐81297, a dopamine D1‐receptor agonist, by alternating light irradiation with two wavelengths.^[^ ^143^ ^]^ Illumination with 365 nm light triggered the trans‐to‐cis isomerization of azobenzene, disrupted the lipid bilayer, and switched on the release within 3 s. The isomerization was reversed, and the release ceased in the presence of 455 nm light (**Figure** **7**A). To simplify the system by using a single stimulus, hybrid magnetic^[^ ^144^ ^]^ or lipidic NPs^[^ ^145^, ^146^ ^]^ reversible thermoresponse have been developed to load chemical modulators for the multiple activations of Ca^2+^ response. Romero and colleagues recently coated MNPs with (poly(oligo(ethylene glycol) methyl ether methacrylate)) (POEGMA) polymer brushes to activate primary rat striatal neurons.^[^ ^144^ ^]^ Dopamine was then absorbed by the MNPs through electrostatic interactions. After exposure to an AMF, the local temperature rose over the low critical solution temperature (LCST) of POEGMA. Consequently, the POEGMA nanocoating collapsed and dopamine was released to stimulate D1 and D2 receptors for Ca^2+^ influx. The reversible thermodynamic phase transition enabled the AMF‐triggered release of dopamine in multiple microdoses, and hence striatal neuronal activity could be repeatedly stimulated for at least three cycles (Figure 7B). Liposomes made from 1,2‐Dipalmitoyl‐sn‐glycero‐3‐phosphocholine (DPPC) undergo phase transition above 43 °C with greatly enhanced permeability. On this basis, Zhen et al. co‐encapsulated capsaicin and semiconducting polymer NPs (SPNs) into PEGylated DPPC liposomes for photothermally‐controlled repetitive release of capsaicin.^[^ ^146^ ^]^ During each cycle of NIR laser irradiation, ≈5–10% capsaicin was released to activate TRPV1 with a 15 s delay, and Ca^2+^ influx could be triggered at least ten times (Figure 7C). In addition to physical encapsulation, chemical modulators can also be released from their precursors. The Bu team recently designed a NO‐generator for activating NO‐sensitive ryanodine receptors (RyRs).^[^ ^147^ ^]^ The caged NO donor molecule cysteine‐NO compound and UCNPs were co‐incorporated into zeolitic imidazolate framework‐8. Under NIR irradiation, UV light emitted by UCNPs cleaved the S—NO bond to liberate NO, leading to S‐nitrosylation of free thiols in RyRs and Ca^2+^ release from ER. The levels of NO remained constant in the absence of NIR light and only rose in its presence, allowing for its multiple releases (Figure 7D).

Despite the merits of the second type of nanotransducers for acute [Ca^2+^]_i_ regulation, chronic stimulation still presents challenges: 1) the irreversible release process and confined loading efficiency of nanotransducers determine their finite stimulation times; 2) the release amount of chemical modulators also gradually decreased in each cycle, making chronic Ca^2+^ stimulation more challenging; 3) the potential premature leakage of chemical modulators results in undesirable stimulation. To circumvent these limitations, the third type of nanotransducers is capable of in situ catalyzing the synthesis of chemical stimulators in the biological microenvironment using self‐supplied constituents, such as oxygen and water. Thus, nanocatalysts including photovoltaic polymer‐based NPs,^[^ ^148^, ^149^ ^]^ photosensitizer oligomer NPs,^[^ ^150^ ^]^ and Pt‐decorated Fe_3_S_4_ nanoclusters^[^ ^26^ ^]^ have been exploited to evoke Ca^2+^ transient. Li et al. designed ROS‐generating oligomer NPs carrying TRPM2‐encoding plasmids for expressing ROS‐sensitive TRPM2 channels and their activation in PC‐3 cancer cells simultaneously.^[^ ^151^ ^]^ π‐conjugated photosensitizer oligomer, when illuminated by NIR light, generated ROS to break Se‐Se bonds and allowed the release of plasmids from disintegrating NPs for enhanced TRPM2 expression. Then, ROS could effectively activate TRPM2 for Ca^2+^ influx. This design, however, is greatly limited to the transfection efficiency of TRPM2. To achieve Ca^2+^ responses in diverse cell types without genetical modifications, our group has recently developed a MOF‐based H_2_O_2_ nanogenerator for photocatalytic activation of IP_3_Rs, which allowed efficient regulation of intracellular Ca^2+^ dynamics and animal behaviors.^[^ ^152^ ^]^ The photocatalyst 9,10‐di(carboxystyryl)anthracene was screened from thirteen small molecules and coordinates to the Zr6 cluster to form MOFs, which solely used O_2_ and water to photosynthesize H_2_O_2_ locally via an oxygen reduction reaction pathway. H_2_O_2_ rapidly activated IP3Rs via thiol oxidation, triggered the release of endogenous Ca^2+^, and stimulated neural activity in the optical tectum of tadpoles and thighs of spinal frogs, thereby eliciting the corresponding motor behaviors (Figure 7E).

## 3 Biomedical Applications of Nanotransducers

**Scheme 4:**
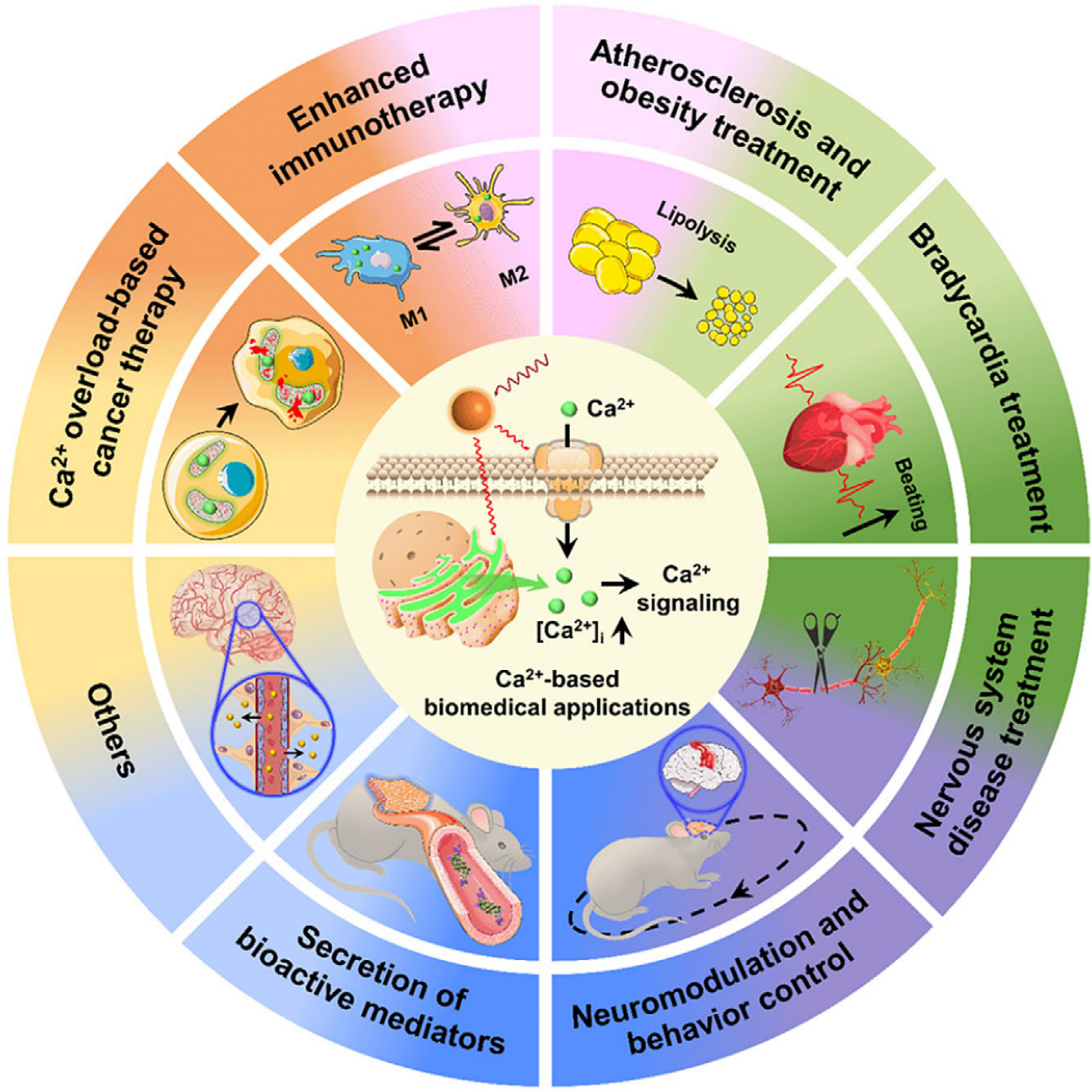
Due to the importance of Ca^2+^ signaling in the regulation of physiological processes, nanotransducers exhibit excellent potential for a wide range of biomedical applications, including disease treatment and behavior control. In these applications, the fundamental working principle of nanotransducers is to manipulate [Ca^2+^]_i_ of specific cells, activate downstream effectors, and cause controlled biological events.

Recent advances in nanotechnology of Ca^2+^ nanotransducers are impacting biomedical applications with the potential to enhance immune system modulation, regulate cellular Ca^2+^‐dependent signaling pathways, differentiation, and apoptosis, and improve the accuracy of neural circuit activation and animal behavior control. In this section, we intend to introduce comprehensively the emerging nanotransducers for improving the therapeutic effects of a series of diseases and advancement of biological understandings (**Scheme** **4**). We highlighted their design mentality, working principle, and application scenario.

### 3.1 Ca^2+^ Overload‐Based Cancer Therapy

Cancer cells maintain a highly precise and intricate Ca^2+^‐regulating system for frequent Ca^2+^ interaction between mitochondria and ER, which is crucial for cancer cells to fulfill their abnormally high energy demands for aberrant proliferation.^[^ ^153^, ^154^, ^155^ ^]^ However, this system also dramatically increases the destructiveness of exogenous Ca^2+^ invasion, making cancer cells more vulnerable to Ca^2+^ overload.^[^ ^156^ ^]^ Cytosolic Ca^2+^ has become an extremely desirable target for the development of novel anticancer drugs.^[^ ^157^ ^]^ Ca^2+^ overload occurs when [Ca^2+^]_i_ surpasses both the buffering capacity of the Ca^2+^‐regulating system and the tolerance level of the cancer cells. Hence, numerous Ca^2+^ nanotransducers have been designed in recent years to achieve Ca^2+^ overload‐induced oncotic therapy via various methods. Specifically, these nanotransducers could induce a sharp increase of [Ca^2+^]_i_ in cancer cells, leading to abnormal mitochondrial Ca^2+^ accumulation and disruption of mitochondrial membrane potential.^[^ ^158^ ^]^ Mitochondrial respiration becomes more robust, resulting in reduced ATP biosynthesis and increased ROS generation.^[^ ^159^, ^160^ ^]^ Reduced ATP production attenuates P‐glycoprotein activity and multi‐drug resistance, and thus improves the chemotherapeutic effect.^[^ ^161^ ^]^ Simultaneously, the mitochondrial membrane damage causes the release of cytochrome C, which activates the caspase cascade family and ultimately leads to cell apoptosis.^[^ ^162^ ^]^ Noticeably, due to the ineffectiveness of monotherapy for tumor eradication, the Ca^2+^ overload method is often combined with other therapies, including hyperthermia, chemotherapy, photodynamic therapy, and increased oxidative stress for more potent therapeutic impacts with less adverse side effects.^[^ ^158^ ^]^

Typically, Ca^2+^ overload could be achieved by using nanomaterials to in situ generate physical stimuli, such as heating, electrical stimulation, and mechanical force, to trigger Ca^2+^ influx or Ca^2+^ release from the endoplasmic reticulum. To elicit a temperature change in tumor cells, a nanotransducer was developed employing doxorubicin (DOX)‐loaded hollow Prussian blue nanocages as the template to coat the polydopamine shell.^[^ ^34^ ^]^ Anti‐TRPV1 antibodies were conjugated to this nanotransducer to boost its activation efficiency. Following binding to TRPV1, this nanotransducer could induce Ca^2+^ influx by photothermal activation of TRPV1. TRPV1‐expressing U373 cells exposed to 808 nm light exhibited a remarkable rise in [Ca^2+^]_i_, enhanced cell‐killing effect, and upregulated caspase‐3 levels, but no significant change was observed in TRPV1‐negative HeLa cells. Meanwhile, other polymer NPs,^[^ ^163^ ^]^ gold nanorods,^[^ ^164^ ^]^ and carbon‐based nanomaterials^[^ ^165^ ^]^ were prepared to induce temperature increases in tumor sites by their intrinsic photothermal conversion property. Owing to the limited tissue penetration of photo‐based stimulation, a series of magnetic field and ultrasound field responding nanomaterials, such as MNPs,^[^ ^166^ ^]^ microbubbles,^[^ ^167^ ^]^ and BaTiO_3_ NPs,^[^ ^111^ ^]^ were designed to introduce heating, mechanical force, and electrical stimulation for inducing Ca^2+^ overload by stimulating the Ca^2+^ channel.

**Figure 8:**
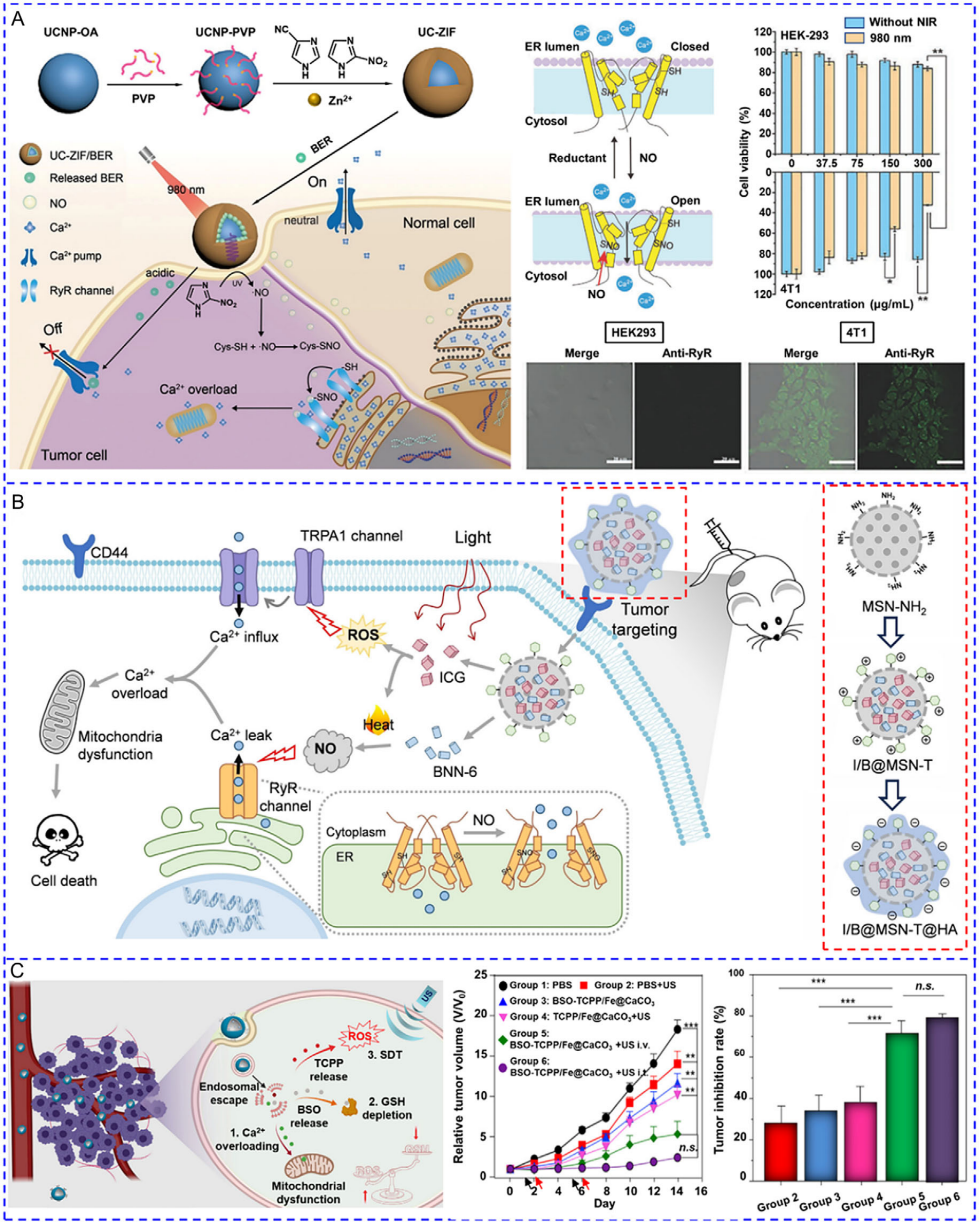
A) NO donor Cys‐SNO and UCNPs were immobilized into a ZIF‐8 MOF. Under NIR irradiation, Cys‐SNO was cleaved by blue light to release NO to activate the RyR receptors. Cell viability and RyR expression exhibit that nanotransducers could kill cancer cells via NO‐mediated RyR activation. Reproduced with permission.^[^ ^172^ ^]^ Copyright 2021, Wiley. B) The thermal‐sensitive NO donor (BNN‐6) and ICG were coloaded into the mesoporous SiO_2_ NPs. The Ca^2+^ nanomodulator generated ROS in response to NIR irradiation, which stimulated the TRPA1 for Ca^2+^ influx from extracellular environments. Concurrently, the converted heat induced the decomposition of BNN‐6 to NO, which would permit the stored Ca^2+^ to leak through RyRs in the ER. Reproduced with permission.^[^ ^168^ ^]^ Copyright 2024, Wiley. C) L‐buthionine sulfoximine (BSO), Fe^2+^, and meso‐tetra‐(4‐carboxyphenyl)porphine (TCPP) were loaded inside PEGylated CaCO_3_ NPs. The constructed nanosystem mediated sonodynamic therapy and Ca^2+^ overload for cancer treatment. In vivo results indicate that the nanosystem significantly inhibited tumor growth. Reproduced with permission.^[^ ^175^ ^]^ Copyright 2020, Cell Press.

Except for physical stimulation, several chemical stimulation strategies have also been proven that the in‐situ generated NO,^[^ ^168^, ^169^ ^]^ ROS,^[^ ^150^ ^]^ or released capsaicin,^[^ ^133^ ^]^ curcumin,^[^ ^170^, ^171^ ^]^ and berbamine^[^ ^172^ ^]^ could also activate Ca^2+^ channels or block Ca^2+^‐efflux pumps to elevate [Ca^2+^]_i_ for cancer cells killing. For example, the Bu group developed a NO nanogenerator to regulate endogenous Ca^2+^ for cancer therapy.^[^ ^172^ ^]^ The upconverted light from UCNPs that were encapsulated inside a zeolitic nitro‐/nitrile‐imidazole framework (ZIF‐8) promoted the NO generation via nitro‐nitrite isomerization and the release of berbamine. NO and berbamine induced Ca^2+^ overload in a synergistic manner: 1) NO efficiently activated the RyRs overexpressed in 4T1 cells, allowing Ca^2+^ to flow out from ER; 2) Berbamine suppressed Ca^2+^ efflux by shutting off Ca^2+^‐excreted pumps in the cell membrane. The viability of normal cells with low RyRs expression was unaffected (**Figure** **8**A). Note that, the efficacy of this approach is highly dependent on the abundance of endogenous Ca^2+^, which may vary among cells. Moreover, RyRs will be closed again after the reduction of SNO by glutathione, and Ca^2+^ will be pumped back into ER by sarcoplasmic reticulum Ca^2+^ ATPase. To achieve an overload of endogenous Ca^2+^‐induced mitochondrial dysfunction, Wu and coworkers developed a cascade release nanoplatform ABT‐99@liposomes/doxorubicin@Fe^III^‐tannic acid, which enabled the directional transport of Ca^2+^ from ER to mitochondria.^[^ ^173^ ^]^ Exposure to NIR light irradiation rapidly released ABT‐199 from the nanoplatform, which combined with ROS to form IP_3_R‐Grp75‐VDAC1 channels between ER and mitochondria and upregulated intramitochondrial Ca^2+^ level, thereby intensifying mitochondrial dysfunction and tumor growth inhibition. To augment Ca^2+^ levels in tumor cells with higher efficiency, the Yoon team devised an NIR‐activatable nanomodulator that utilized dual‐source endogenous Ca^2+^.^[^ ^168^ ^]^ In this nanomodulator, a thermal‐sensitive NO donor (BNN6) and ICG were coloaded into the mesoporous SiO_2_ NPs, and they were further coated with hyaluronic acid as the gatekeeper and tumor targeting unit. The Ca^2+^ nanomodulator generated ROS in response to NIR irradiation, which stimulated the TRPA1 to facilitate Ca^2+^ influx from extracellular environments. Concurrently, the generated heat induced the BNN6 decomposition into NO, which permit the stored Ca^2+^ to leak through RyRs in the ER (Figure 8B).

The direct delivery of exogenous Ca^2+^ to the cytoplasm provides a more straightforward alternative for inducing Ca^2+^ overload. Among them, calcium‐based nanomaterials due to their excellent biocompatibility, biodegradability, low cytotoxicity, pH‐responsiveness, and low environmental risk, have drawn considerable attention in the past decades as drug delivery vehicles. However, their significance in Ca^2+^ overload has only lately been discovered due to the growing comprehension of cell metabolism and physiology. Considering the high Ca^2+^ density stored in the solid NPs, recent studies have demonstrated that their overdosing can directly and potently induce Ca^2+^ overload in cancer cells. As aforementioned, a variety of calcium‐based nanomaterials including CaF_2_,^[^ ^127^ ^]^ CaCO_3_,^[^ ^128^ ^]^ CaO_2_,^[^ ^129^ ^]^ Ca_3_(PO_4_)_2_,^[^ ^130^ ^]^ and Ca_2_SiO_4_^[^ ^131^ ^]^ NPs, have been synthesized. Calcium‐based nanomaterials are endocytosed and transported to the acidic endo/lysosome in cancer cells, where they are degraded to liberate Ca^2+^ ions. The rapidly increasing osmotic pressure causes the endo/lysosome to rupture, allowing Ca^2+^ to leak into the cytoplasm.^[^ ^174^ ^]^ Dong et al. utilized the biocompatible CaCO_3_ NPs to co‐encapsulate meso‐tetra‐(4‐carboxyphenyl)porphine (TCPP), ferric ion, and L‐buthionine sulfoximine (BSO) for constructing the pH‐dissociable hollow coordination nanostructures.^[^ ^175^ ^]^ The obtained TCPP‐Fe@CaCO_3_ could perform Ca^2+^ overload‐enhanced photodynamic and chemodynamic therapy. As revealed in Figure 8C, the nanocomplexes dissociated in the endo/lysosome to release BSO for inhibition of glutathione (GSH) biosynthesis. On the promotions of Ca^2+^‐overload and decreased GSH levels, TCPP‐based photodynamic therapy and ferric ion‐mediated chemodynamic therapy could be markedly augmented. Of note, due to the high stability of CaCO_3_, the Ca^2+^ generation rate is not satisfactory. To boost Ca^2+^ release, Shen et al. developed a smart PEGylated nanosystem by coating CaO_2_ NPs with Fe‐contained ZIF.^[^ ^176^ ^]^ To achieve photodynamic therapy and increase blood circulation time, the nanocomplex (CaZFCP) was further modified by Ce6 and PEG. In this study, CaZFCP decomposed to achieve Ca^2+^‐overload and H_2_O_2_/O_2_ self‐supply under the weakly acidic TME. The generated H_2_O_2_ and O_2_ significantly enhanced Fe‐based chemodynamic therapy and Ce6‐mediated photodynamic therapy, respectively. Highly reactive CaO_2_‐based nanogenerators must be handled with caution and kept away from water during storage. They would also rapidly react with biological fluid in the bloodstream and break down prior to reaching target cells.

**Figure 9:**
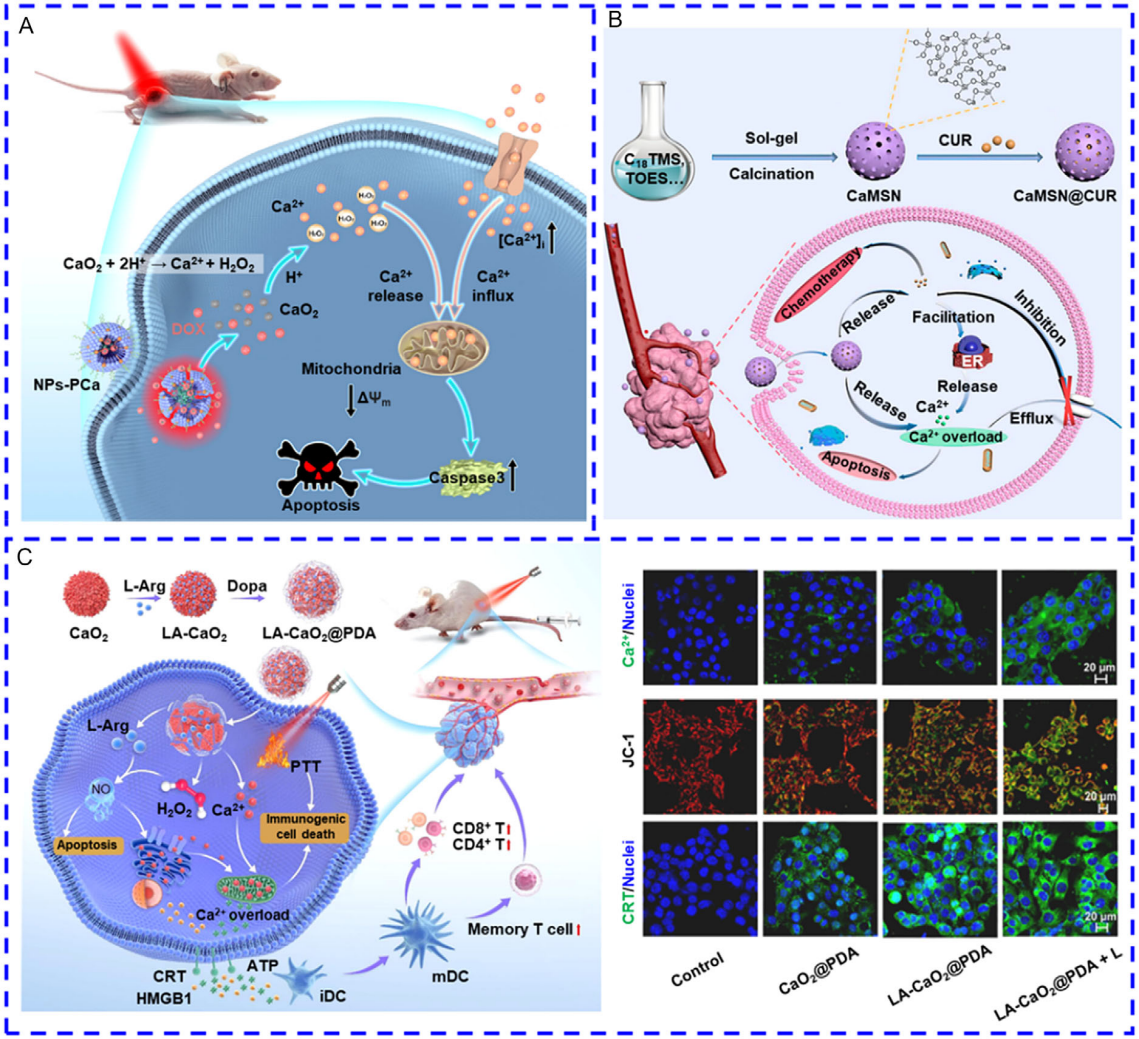
A) Photothermal polymer was used to prepare CaO_2_ for loading DOX. Upon endocytosed by cancer cells, the release of Ca^2+^ and polymer‐induced photothermal effect simultaneously increased [Ca^2+^]_i_ to induce Ca^2+^ overload. Reproduced with permission.^[^ ^177^ ^]^ Copyright 2022, American Chemical Society. B) Ca‐doped mesoporous silica NPs with co‐loaded curcumin could induce Ca^2+^ overload by facilitating Ca^2+^ release and suppressing Ca^2+^ efflux. Reproduced with permission.^[^ ^170^ ^]^ Copyright 2022, American Chemical Society. C) CaO_2_ loaded with L‐arginine and coated by polydopamine was a promising nanotransducer for elevating [Ca^2+^]_i_ via releasing Ca^2+^ from CaO_2_ and inducing Ca^2+^ efflux from the endoplasmic reticulum by biosynthesized NO. The [Ca^2+^]_i_, mitochondrial membrane potential, and surface CRT exposure are demonstrated. Reproduced with permission.^[^ ^178^ ^]^ Copyright 2022, Elsevier.

As described above, there are three strategies to induce Ca^2+^ overload for cancer therapy, namely the introduction of physical stimuli to activate Ca^2+^ channels, the in situ release/generation of chemical modulators to induce cytosolic Ca^2+^ accumulation, and the use of calcium‐based nanomaterials to directly transport Ca^2+^ to the cytoplasm. However, due to the inability of [Ca^2+^]_i_ to reach the lethal level, their anticancer effect is insignificant. To further improve the efficiency of Ca^2+^ overload, growing efforts are focused on the development of nanotransducers that combine two or three strategies in a single system to achieve ultrahigh‐effective Ca^2+^ overload. Typically, these nanotransducers were designed by loading physical convertors or chemical modulators in calcium‐based nanomaterials. Zhou et al. recently prepared NIR light‐responsive conjugated polymer NPs, which concurrently facilitated Ca^2+^ inflow and Ca^2+^ release.^[^ ^177^ ^]^ The nanotransducer was formed by using DOX‐loaded CaO_2_ nanoparticles as the core and coating with DPPC‐DSPE‐PEG2000‐NH_2_ and PDPP. CaO_2_ could be degraded to liberate Ca^2+^, while photothermal heating of the nanotransducer raised the temperature upon illumination with 808 nm light, thereby activating TRPV1 to cause Ca^2+^ influx (**Figure** **9**A). Recently, Hu et al. developed a multifunctional nanosystem that could simultaneously induce Ca^2+^ overload by multiple pathways and produce chemotherapy for synergistic tumor therapy.^[^ ^170^ ^]^ The nanosystem (CaMSN@CUR) was prepared by synthesizing a Ca‐doped mesoporous silica nanoparticle (CaMSN), followed by loading them with the anticancer drug curcumin. CaMSN served as the Ca^2+^ generator, whereas curcumin not only exhibited a chemotherapeutic effect but also facilitated Ca^2+^ release from ER to the cytoplasm and inhibited Ca^2+^ efflux out of cells (Figure 9B). Except for delivering chemical modulators to achieve co‐stimulation of Ca^2+^ overload with calcium‐based nanomaterials, in situ generation of chemical modulators by the TME has the potential to improve the efficiency of tumor‐specific therapy efficacy. Hao et al. used mesoporous CaO_2_ to load and oxidize L‐arginine to achieve a controllable local release of NO in the acidic TME.^[^ ^178^ ^]^ After being coated by polydopamine, this NP significantly inhibited tumor growth by the combination of polydopamine‐mediated photothermal therapy, and CaO_2_ and NO co‐triggered Ca^2+^ overload (Figure 9C). Compared to monotherapy by CaO_2_ alone, cooperation of CaO_2_ and NO caused much higher [Ca^2+^]_i_, more surface calreticulin (CRT) exposure, and more declined mitochondrial membrane potential.

### 3.2 Nanotransducer‐Enhanced Immunotherapy

**Figure 10:**
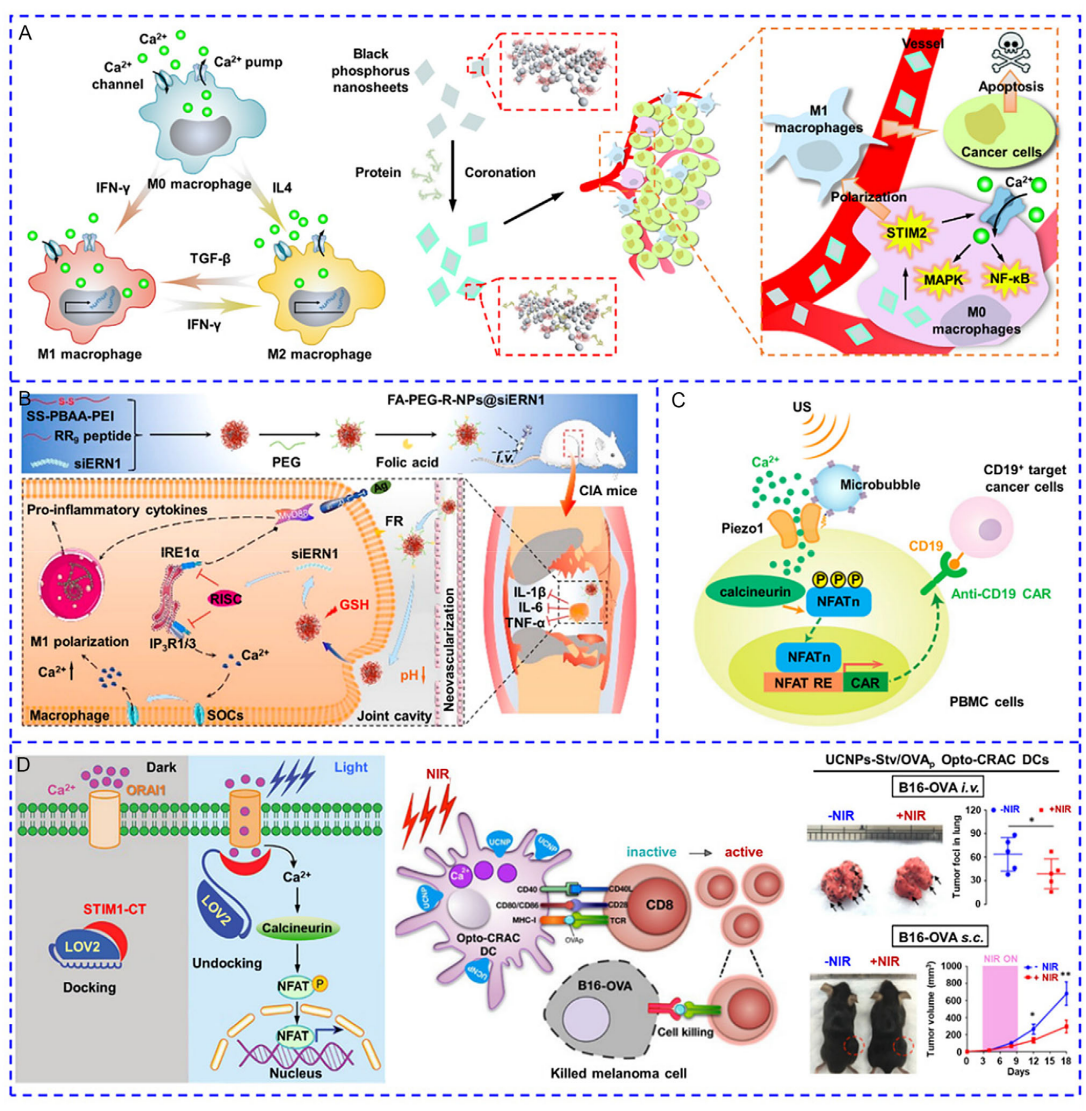
A) The basic principle of using nanotransducers to regulate macrophage polarization. Block phosphorus‐corona complexes interacted with calmodulin to induce the Ca^2+^ influx, which could polarize M0 macrophages to the M1 phenotype. Reproduced with permission.^[^ ^180^ ^]^ Copyright 2020, Royal Society of Chemistry. B) Polymer NP loaded with siRNA was synthesized to target the nucleus signaling 1 gene in the endoplasmic reticulum. siRNA inhibited the expression of IP_3_R1/3 and thus reduced [Ca^2+^]_i_, which further induced the M2 polarization. Reproduced with permission.^[^ ^183^ ^]^ Copyright 2021, American Chemical Society. C) Streptavidin‐modified microbubble amplified the impact of ultrasound and activated Piezo1 to induce Ca^2+^ influx, which promoted the expression of chimeric antigen receptor (CAR) protein expression. Reproduced with permission.^[^ ^167^ ^]^ Copyright 2018, United States National Academy of Sciences. D) Schematic diagram of using light to induce Ca^2+^ influx by Opto‐CRAC toolkits. The basic principle of using UCNPs and Opto‐CRAC to modulate the immune response and inhibit tumor growth is demonstrated. Reproduced with permission.^[^ ^184^ ^]^ Copyright 2015, eLife Sciences Publications.

Ca^2+^ tightly regulates the immune response of immune cells in terms of their metabolism, proliferation, polarization, differentiation, and secretion of cytokines.^[^ ^179^ ^]^ Therefore, the development of nanotransducers to regulate the host immune system by manipulating [Ca^2+^]_i_ of immune cells has gained a certain degree of attention in recent years. Especially, using nanotransducers to control macrophage polarization has drawn extensive research interest because macrophages are essential for innate immunity.^[^ ^180^ ^]^ M1 macrophages secrete proinflammatory cytokines with potent killing effects on malignant cells and invading pathogens, whereas anti‐inflammatory M2 macrophages exert an immune suppressive effect, promoting tumor progression and tissue repair.^[^ ^181^ ^]^ It is generally established that an increase in [Ca^2+^]_i_ promotes uncommitted (M0) macrophage polarization toward the M1 phenotype. In contrast, lowering [Ca^2+^]_i_ favors polarization toward the M2 anti‐inflammatory phenotype.^[^ ^182^ ^]^ Thus, over the past 5 years, several nanotransducers have been developed to regulate [Ca^2+^]_i_ not only for directing the polarization of M0 macrophage into M1/M2 macrophage but also for affecting the interconversion between the M1/M2 phenotypes (**Figure** **10**A). To facilitate M1 polarization, black phosphorus nanosheets with adsorbed protein corona were used to interact with calmodulin to activate stromal interaction molecule 2 (STIM2) for accelerating Ca^2+^ influx in macrophages.^[^ ^180^ ^]^ Owing to the elevated cytosolic Ca^2+^ level, the p38 and nuclear factor (NF)‐κB of M0 macrophages were activated, and M0 macrophages were polarized to the M1 phenotype. For the treatment of autoimmune inflammatory diseases, Feng et al. developed an endoplasmic reticulum to nucleus signaling 1 interfering RNA (siERN1) nanoprodrug to control macrophage M2 polarization and block toll‐like receptors signaling by interfering with the activity of IP_3_R1.^[^ ^183^ ^]^ Polyethylene glycol and polyethyleneimine were introduced to construct a nanocarrier to improve biocompatibility and facilitate lysosome escape, respectively. The siERN1‐loaded nanocarriers were phagocytosed by macrophages and then downregulated the expression of ERN1 and IP_3_R1 to decrease [Ca^2+^]_i_ (Figure 10B).

In addition to regulating macrophage polarization, nanotransducers have also been designed to regulate the function of T lymphocytes^[^ ^167^ ^]^ and dendritic cells^[^ ^184^, ^185^ ^]^ via controlling gene expression. Chimeric antigen receptor (CAR) T‐cell therapy is a paradigm‐shifting therapeutic approach for cancer immunotherapy.^[^ ^186^ ^]^ Pan et al. engineered primary T cells with genetic circuits that were able to transform ultrasound waves into transcriptional activation for CAR expression.^[^ ^167^ ^]^ Integrin‐targeting microbubbles adhering to the cell membrane amplified the impact of low‐frequency (2 MHz) ultrasound and activated Piezo1 to induce Ca^2+^ influx, which ultimately promoted CAR protein expression for recognizing, attaching, and destroying CD19‐antigen expressing B‐cell leukemia cells (Figure 1C). Ca^2+^ entry through store‐operated Ca^2+^ release‐activated Ca^2+^ (CRAC) channels is of vital importance to replenish ER Ca^2+^ when they are depleted. Based on this biological mechanism, He et al. integrated UCNPs and opto‐CRAC toolkits to establish a NIR light‐stimulated platform to induce Ca^2+^ influx from CRAC channels, which further activated Ca^2+^/nuclear factor of activated T (NFAT) cells pathway in T lymphocyte, macrophages, and dendritic cells.^[^ ^184^ ^]^ ORAI1StrepTag and LOVSoc (stromal interaction molecule 1 cytosolic domain, STIM1‐CT fragments fused with a photo‐switch LOV2 domain) were genetically encoded into immune cells. By docking toward the LOV2 domain in the dark, STIM1‐CT remained inactive. Upon NIR irradiation, STIM1‐CT was unleashed and moved toward the plasma membrane to directly open ORAI1 (the pore‐forming subunit of CRAC channel) Ca^2+^ channel, initiating Ca^2+^ influx (Figure 10D). In vitro, photoirradiation boosted the cytokine production in naïve CD4 + T cells and amplified the inflammasome activation in THP‐1‐derived macrophages. In vivo, dendritic cell‐mediated immunotherapy in the B16‐ovalbumin murine melanoma model was demonstrated. Bone marrow‐derived dendritic cells were first genetically modified to express light‐sensitive Opto‐CRAC and loaded with UCNPs/ovalbumin, and then intravenously injected into mice. NIR light caused the maturation of dendritic cells, boosting ovalbumin antigen presentation and CD8 + T cell priming and activation, which effectively eradicated melanoma cells at distal sites.

### 3.3 Treatment of Atherosclerosis and Obesity

**Figure 11:**
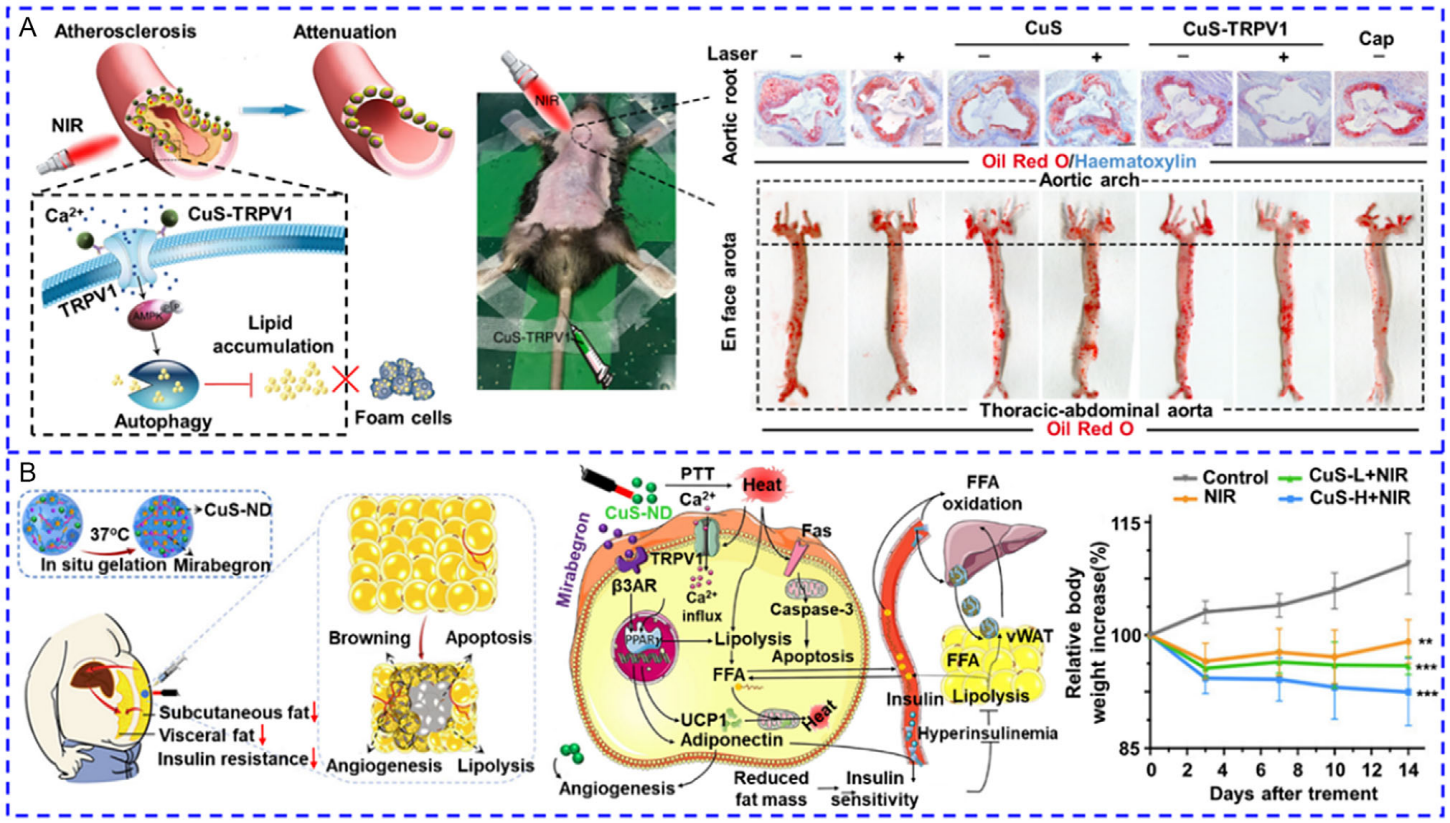
A) Anti‐TRPV1 antibody‐modified CuS NPs efficiently activated TRPV1 of vascular smooth muscle cells to induce their autophagy and prevent lipid accumulation. The photograph shows the Oil Red O‐stained aortic root sections of rats which were treated by different therapeutics. Reproduced with permission.^[^ ^29^ ^]^ Copyright 2018, Springer Nature. B) CuS NPs could convert brown adipose tissue to white adipose tissue. The Ca^2+^ signaling pathway activated by CuS through a photothermal effect and the relative body weight increase of different treatment groups are demonstrated. Reproduced with permission.^[^ ^189^ ^]^ Copyright 2022, American Chemical Society.

Although atherosclerosis and obesity are two different diseases, it is generally considered that both are caused by the excessive deposition of cholesterol in atherosclerosis and lipids and specific triglycerides in adipose tissue.^[^ ^187^ ^]^ Unluckily, the role of [Ca^2+^]_i_ in the regulation of lipid accumulation remains unclear. Even so, there are some efforts using nanotransducers to realize Ca^2+^‐mediated alleviation of the symptoms of arterial atherosclerosis and obesity. Recently, an anti‐TRPV1 antibody‐modified CuS NP was designed to activate TRPV1 channels on the plasma membrane of vascular smooth muscle cells (VSMCs).^[^ ^29^ ^]^ In this design, the excellent photothermal property of CuS could remotely control the activation of TRPV1 under 980 nm NIR irradiation, thereby initiating Ca^2+^ influx. The increased [Ca^2+^]_i_ could activate autophagy in oxidized low‐density lipoprotein‐treated VSMCs. Autophagy activation enhanced ATP‐binding cassette transporter A1 (ABCA1)‐mediated cholesterol efflux and inhibited foam cell formation (**Figure** **11**A). After 12 weeks of treatment, CuS NPs significantly reduced lipid storage and atherosclerotic lesions in mice fed a high‐fat diet. Similar design concepts have also been applied to obesity treatment. White adipose tissue (WAT) can affect body fat metabolism by depositing triglycerides for energy storage and secreting adipokines, whereas brown adipose tissue (BAT) utilizes glucose and free fatty acids as an energy source to produce heat and reduce obesity.^[^ ^188^ ^]^ Although WAT is almost absent in adults, brown adipocytes can be produced by browning white adipocytes. Zan et al. incorporated CuS nanodots and mirabegron, an emerging diet pill, into the hydrogel to treat mice fed with a high‐fat diet via photothermal‐mediated WAT remodeling and enhancing whole‐body metabolism (Figure 11B).^[^ ^189^ ^]^ Photothermal heating of CuS nanodots activated TRPV1‐gated Ca^2+^ influx, which in turn activated peroxisome proliferator‐activated receptor γ receptors in the nucleus of adipocytes and ultimately induced lipolysis and browning by enhancing the expression of lipases and uncoupling protein 1 (UCP1). The synergistic treatment of CuS nanodot‐based photothermal therapy and mirabegron‐mediated chemotherapy dramatically blocked the obesity process during the high‐fat feeding period.

### 3.4 Treatment of Bradycardia

Slow heart rate, known as bradycardia, is a type of abnormal heart rhythm (arrhythmia) controlled by electrical signals.^[^ ^190^ ^]^ Although electronic pacemakers are widely used in clinical treatment by enhancing electrical conduction in hearts, their invasive implantation increases the risk of infection.^[^ ^191^ ^]^ Considering the key role of Ca^2+^ in excitation–contraction coupling and electric rhythms in cardiac function, optogenetic control of cardiomyocyte beating has been demonstrated to induce cation influx and membrane depolarization by expressing photosensitive cation channels such as ChR2 on the cell membrane.^[^ ^192^, ^193^ ^]^ Due to the necessity for viral infection, the clinical implementation of this approach, despite being effective and non‐invasive, confronts significant obstacles.

**Figure 12:**
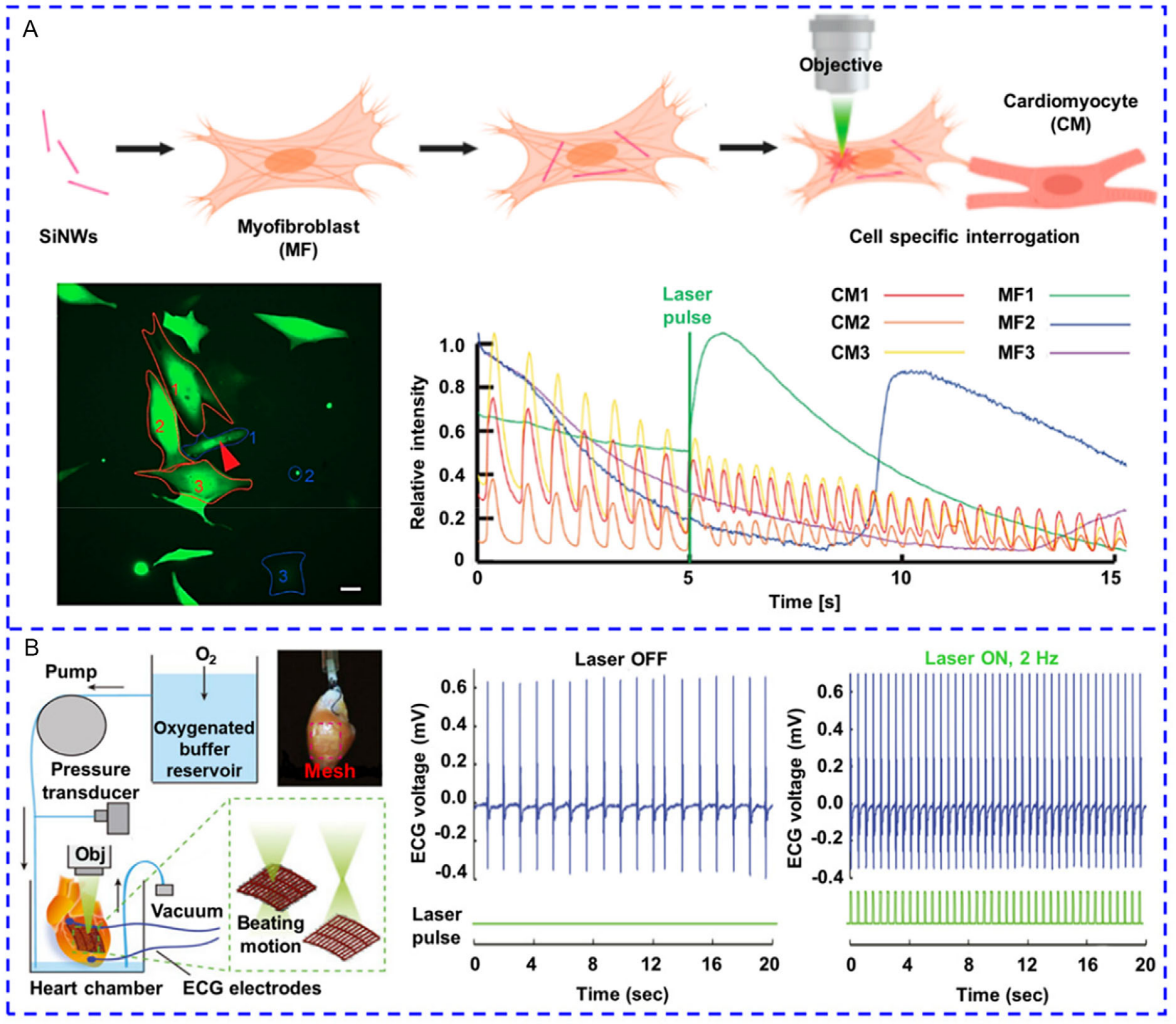
A) Schematic diagram of myofibroblasts with internalized silicon nanowires myofibroblasts to alter the contraction of cocultured cardiomyocytes. The contraction rates of cocultured cardiomyocytes (with the red lines) indicated by Fluo‐4 intensities were affected after the stimulation of neighboring cardiomyocytes (indicated by the red arrow). Reproduced with permission.^[^ ^195^ ^]^ Copyright 2020, American Chemical Society. B) The device for assessing optical stimulation of the ex vivo rat hearts. Electrocardiogram recordings from the heart covered with a SU‐8/PIN‐SiNW mesh, before (left) and during (right) stimulation with 532 nm laser pulses at 2 Hz, are demonstrated. Reproduced with permission.^[^ ^196^ ^]^ Copyright 2019, United States National Academy of Sciences.

Recently, nanotransducers have been explored as a means of avoiding viral infection by converting optical signals to thermal/electrical signals to directly regulate [Ca^2+^]_i_. As an example, Gentemann et al. increased the contraction rate of neonatal rat cardiomyocytes by irradiating Au NPs with ps‐pulsed light.^[^ ^194^ ^]^ Photothermal heating of Au NPs transiently permeabilized their anchored cell membrane by forming plasmonic nanobubbles. Ca^2+^ inflow boosted the beating rate of cardiomyocytes by 57% within 15 s before gradually decreasing. Notably, the human heart comprises multiple cell types, making it challenging to precisely activate a particular cell type (e.g., cardiomyocyte). However, because of the intercellular connections, Ca^2+^ can directly diffuse from non‐excitable cells into cardiomyocytes across gap junctions and affect the electrical activity of cardiomyocytes.^[^ ^193^ ^]^ This “tandem cell unit” concept was recently demonstrated by the photostimulation of semiconducting silicon nanowires within myofibroblasts to alter the contraction of cocultured cardiomyocytes (**Figure** **12**A).^[^ ^195^ ^]^ Ca^2+^ was released from intracellular Ca^2+^ stores in myofibroblasts by both photoelectrochemical and photothermal mechanisms, and Ca^2+^ waves were immediately propagated to neighboring cardiomyocytes, increasing resting membrane potential and contraction rate. As both direct and indirect stimulation of cardiomyocytes proved effective, all cells in ex vivo rat hearts were stimulated simultaneously to resynchronize the heartbeat to the desired frequency.^[^ ^196^ ^]^ In this study, randomly oriented silicon nanowires were incorporated into a polymer mesh, which was adhered to the myocardium of the ex vivo rat heart without additional adhesive (Figure 12B). After 5 min of exposure to pulsed light, the inherent beating frequency of 0.9 Hz was photo‐stimulated to the targeted 2 Hz without causing tissue damage.

### 3.5 Treatment of Nervous System Disease

**Figure 13:**
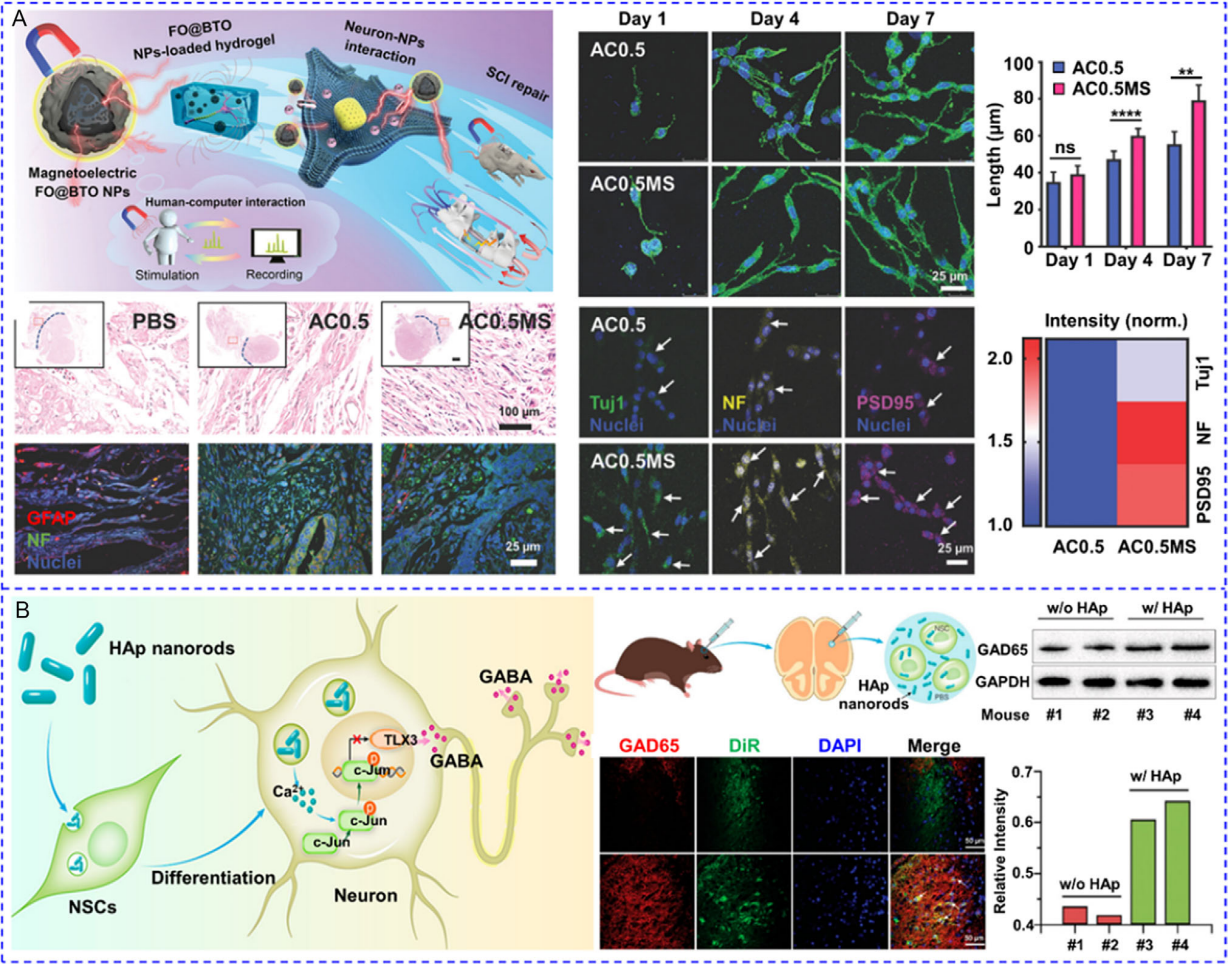
A) Core/shell structured Fe_3_O_4_@BaTiO_3_ NPs generated electrical stimuli under the magnetic field control to manipulate [Ca^2+^]_i_. The axon length and expression of neurogenic proteins of PC‐12 cells significantly increased after electrical stimuli. H&E staining and immunofluorescence images showed more infiltrated cells and fewer cavities after treatment in spinal cord cross‐sections of the hemi‐transected SCI rat model. Reproduced with permission.^[^ ^198^ ^]^ Copyright 2021, Wiley. B) Working principle of hydroxyapatite (HAp) nanorods for promoting neural differentiation of neural stem cells (NSCs) into GABAergic neurons. NSCs with HAp nanorods were transplanted into the cortex of the mouse. HAp nanorods upregulated both the gene transcription and protein expression of GAD65 in the brain slices. Reproduced with permission.^[^ ^200^ ^]^ Copyright 2021, American Chemical Society.

In recent years, great efforts have been focused on the development of nanotransducers for remote, spatiotemporal, and precise manipulation of [Ca^2+^]_i_ of neurons for the treatment of nervous system diseases, such as nerve injury,^[^ ^147^, ^197^, ^198^ ^]^ Parkinson's disease,^[^ ^30^, ^101^, ^104^, ^105^ ^]^ depression,^[^ ^95^ ^]^ and epilepsy.^[^ ^113^ ^]^ It has been demonstrated that local [Ca^2+^]_i_ manipulation by nanotransducers accelerates neurite outgrowth and nerve transmission, indicating great potential for the treatment of nerve injuries, such as spinal cord injury (SCI), peripheral nerve damage, and traumatic brain injury. For example, Zhang et al. designed an implantable hyaluronan/collagen hydrogel that was loaded with core/shell magnetoelectric Fe_3_O_4_@BaTiO_3_ NPs to deliver electrical stimulation to impaired neurons (**Figure** **13**A).^[^ ^198^ ^]^ PC‐12 cells exposed to a pulsed magnetic field generated electricity that upregulated the expression of VGCC. Ca^2+^ influx through VGCC greatly enhanced axon elongation and expression of neurogenic proteins. In the hemi‐transected SCI rat model, the treated groups exhibited more cell infiltration and fewer voids at the injury site. Neuronal differentiation of neural stem cells (NSCs) is a feasible alternative treatment for SCI. However, chronic activation of immune cells at the injury site leads to secondary neurological lesions and creates an immune microenvironment hostile to NSC differentiation. Zhu and colleagues have recently synthesized Mg/Al layered double hydroxide NPs (Mg/Al‐LDH) to accelerate NSCs migration, neural differentiation, L‐type Ca^2+^ channel activation, and action potential generation.^[^ ^199^ ^]^ In vivo experiments revealed that Mg/Al‐LDH dramatically improved motor evoked potential, demonstrating their capacity for SCI recovery. Ca^2+^‐driven differentiation of NSCs can also be applied to regenerate other neuron types.^[^ ^119^, ^200^ ^]^ For instance, Shen et al. produced hydroxyapatite nanorods to induce NSCs differentiation for the production of γ‐aminobutyric acid (GABA), a primary inhibitory neurotransmitter (Figure 13B).^[^ ^200^ ^]^ Lysosomal degradation of hydroxyapatite (HAp) nanorods significantly raised [Ca^2+^]_i_, thereby activating c‐Jun and inhibiting TLX3, a gene involved in GABAergic/glutamatergic selection. Transplantation of NSCs and HAp nanorods into the cortex of the mouse led to a substantial increase in both the mRNA and protein expression levels of GAD65, an enzyme responsible for GABA synthesis, within the brain transplant area. The preliminary evidence show that HAp nanorods could promote the regeneration of GABAergic neurons.

Parkinson's disease (PD) is the second most common neurodegenerative disorder in humans. The pathogenesis of PD starts from the nuclear gene mutation of dopaminergic neurons, followed by the inhibition of the hydrolysis of α‐synuclein, promotion of its aggregation in mitochondria, and accumulation of intraneuronal Lewy bodies. This leads to the production of excessive ROS and degeneration of dopaminergic neurons, thereby downregulating the expression of tyrosine hydroxylase (TH) and reducing the secretion of dopamine.^[^ ^201^, ^202^, ^203^ ^]^ Clinically, deep brain stimulation (DBS) by electrode implantation into the subthalamic nucleus could electrically stimulate nervous systems and was superior to medical therapy in alleviating PD and early motor complications.^[^ ^204^ ^]^ Nonetheless, the mechanisms underlying its symptom‐alleviating effects are still not entirely understood. As alternatives to invasive DBS, several studies indicate that adjusting [Ca^2+^]_i_ in dopaminergic neurons^[^ ^101^, ^104^, ^105^, ^118^, ^205^ ^]^ by nanotransducers can ameliorate disease progression, which may provide new therapeutic implications for PD.

**Figure 14:**
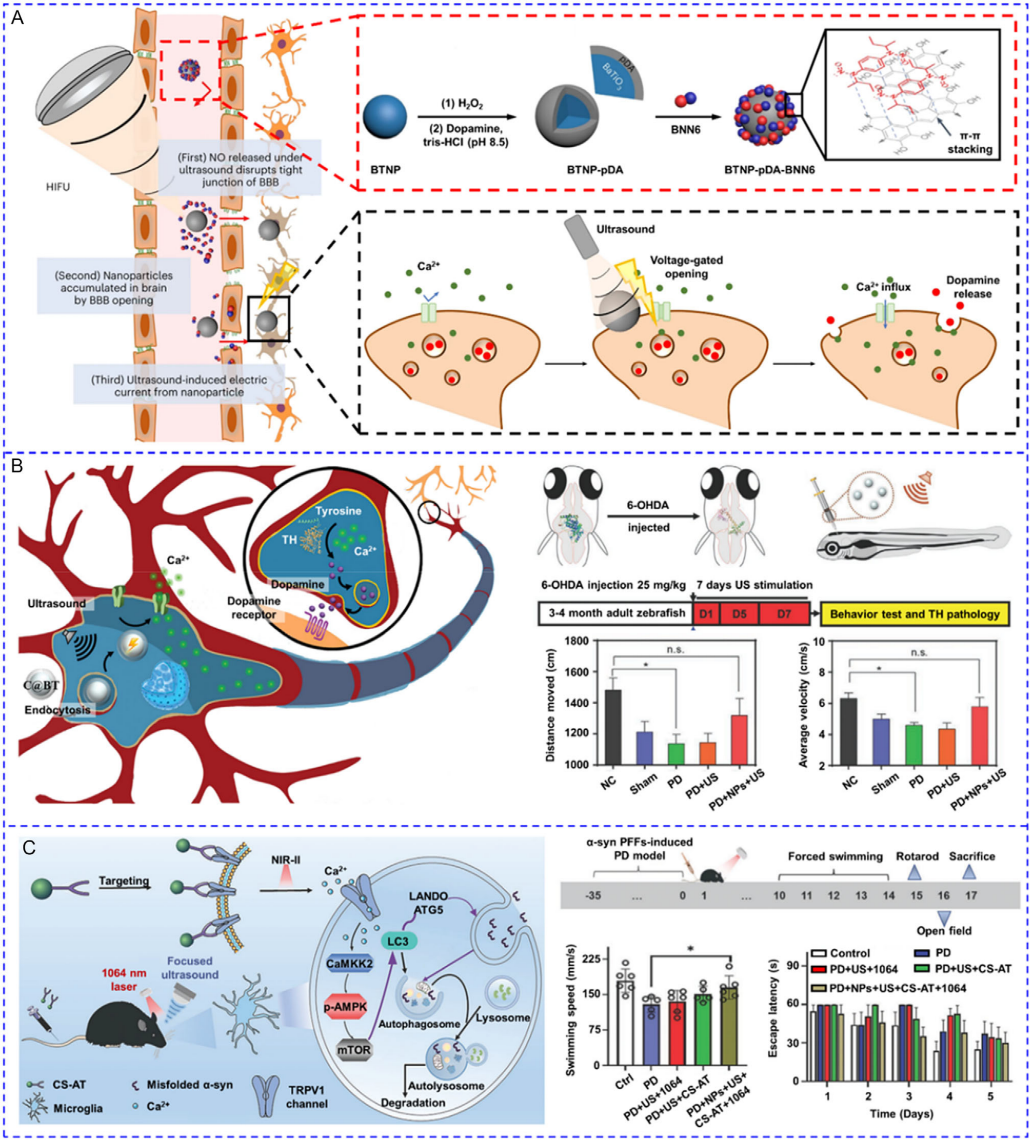
A) Piezoelectrically induced output current could directly restore the dysfunctional neuronal circuits in the mouse Parkinson's disease model. Reproduced with permission.^[^ ^101^ ^]^ Copyright 2023, Springer Nature. B) C@BT core–shell NPs can electrically stimulate Ca^2+^ influx under ultrasound treatment. The stimulated cells secreted dopamine to enhance the plasticity of neurons. The distance and velocity of adult zebrafish increased after treatment. Reproduced with permission.^[^ ^104^ ^]^ Copyright 2020, Wiley. C) Cu_2−*x*_Se NPs were conjugated by anti‐TRPV1 antibodies and then used to stimulate TRPV1 under focused ultrasound irradiation. Swimming speed and the escape latencies recovered for the rat treated by nanotransducers and ultrasound‐irradiated nanotransducers. Reproduced with permission.^[^ ^30^ ^]^ Copyright 2022, Wiley.

In this part, we provide several representative examples reflecting the advanced developments of piezoelectrical nanotransducers,^[^ ^101^, ^104^, ^105^ ^]^ magnetothermal NPs,^[^ ^205^ ^]^ and nanoscale optoelectrodes^[^ ^118^ ^]^ to alleviate the symptoms of PD. To reverse the motor deficit, Hescham et al. used magnetothermal nanotransducers to stimulate the subthalamic nucleus in both mild and severe mouse PD models.^[^ ^205^ ^]^ After viral infection to express TRPV1, MNPs were directly injected into the subthalamic nucleus. Despite 40% (mild model) or 90% (severe model) loss of tyrosine hydroxylase positive cells, magnetothermal heating by an AMF effectively activated neuronal circuits in PD mice to alleviate motor disability. Of note, this strategy treats the symptoms rather than eliminating the causes. By applying piezoelectrically induced output current, the Kim team directly stimulated dopamine release from dopaminergic neurons located deep inside the brain to restore the dysfunctional neuronal circuits.^[^ ^101^ ^]^ Following systemic administration, BNN6 absorbed on the piezoelectric NPs could release NO under high‐intensity focused ultrasound (HIFU). This caused a transient disruption of the tight junction in the BBB, which enabled the accumulation of NPs into the brain parenchyma. Piezoelectrical NPs not only activated dopaminergic neurons under HIFU to release dopamine, but also increased the number of TH^+^ neurons, thereby ameliorating the symptoms of mouse Parkinson's disease model (**Figure** **14**A). The Shen group further confirmed that electric stimulation could modulate neural plasticity and reverse dopaminergic neuron dysfunction.^[^ ^104^, ^105^ ^]^ One of their work involved the fabrication of a carbon shell‐coated piezoelectric BaTiO_3_ nanotransducer (C@BT) with improved ultrasound absorption and electromagnetic field generation.^[^ ^104^ ^]^ A rapid elevation in [Ca^2+^]_i_ in response to acoustic stimulation resulted in dramatic overexpression of both synaptophysin (an indicator for synaptic plasticity) and tyrosine hydroxylase (a rate‐limiting factor in the biosynthesis of dopamine) in neurons (Figure 14B). In the open‐field test, PD zebrafish treated with C@BT and ultrasound demonstrated remarkable restoration of movement, indicating ameliorated neural behavioral disorders.

It is proposed that misfolded α‐synuclein released from donor cells can be transferred to neighboring neurons; as seeds, they further induce the misfolding and aggregation of more α‐synuclein.^[^ ^206^ ^]^ Therefore, facilitating clearance of intraneuronal α‐synuclein by microglia is supposed to halt Parkinson's progression. For this, Yuan et al. used TRPV1‐modified Cu_2−*x*_Se nanodots to target TRPV1‐expressing microglia and activated their Ca^2+^ signaling pathway to promote their phagocytosis (Figure 14C).^[^ ^30^ ^]^ In the α‐synuclein preformed fibrils‐induced PD mouse model, nanodots were injected intravenously and delivered to the striatum in the brain using focused ultrasound to temporarily open the BBB. Ca^2+^ influx through TRPV1 channels was facilitated by 1064 nm laser irradiation, which activated the ATG5 and Ca^2+^/CaMKK2/AMPK/mTOR signaling cascade, thereby enhancing the autophagic destruction of α‐synuclein. In the forced swimming, rotarod, and open field tests, photothermal heating restored motor ability and ameliorated anxiety and memory dysfunction in PD mice.

### 3.6 Neuromodulation for Animal Behavior Control

Numerous nanotransducers have been reported to selectively modulate particular neuron subtypes by converting external energy to physical or chemical outputs for dissecting functional neural circuits and controlling their activities. The working principle of nanotransducer‐enabled [Ca^2+^]_i_ regulation for stimulating neural circuits is that an increase in [Ca^2+^]_i_ elicits action potential trains in neurons, which travel along the axon and trigger neurotransmitter release for neural communication and regulation of animal behaviors.^[^ ^36^, ^207^ ^]^ Hence, nanotransducers as effective tools in the field of neuromodulation have been applied to uncover the functional connectivity in brain circuits, for example, how neural circuits generate/regulate behavior.

**Figure 15:**
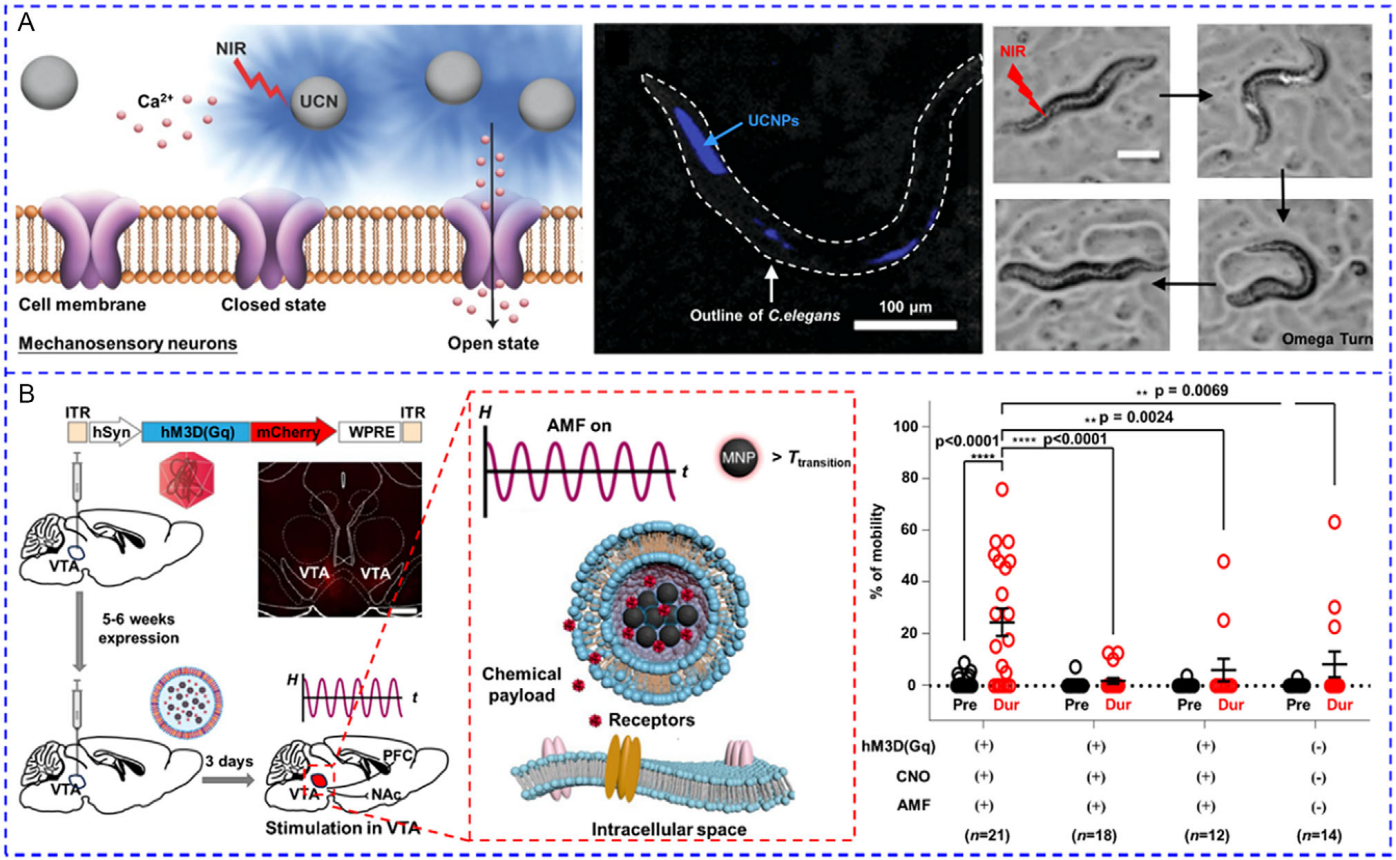
A) *C.elegans* with UCNPs exhibited a drastic change in direction of movement akin to being touched upon exposure to 980 nm. Reproduced with permission.^[^ ^59^ ^]^ Copyright 2016, Wiley. B) AMF‐triggered release of CNO from magnetoliposomes to modulate neural circuits in the VTA and exhibited enhanced swimming. Reproduced with permission.^[^ ^145^ ^]^ Copyright 2019, Springer Nature.

Due to a great understanding of complex neural circuitry and its correlation with specific behaviors, nanotransducers can manipulate [Ca^2+^]_i_ for precise and remote control of different behaviors in diverse animals including *Caenorhabditis elegans* (*C. elegans*),^[^ ^36^ ^]^ *Hydra vulgaris*,^[^ ^208^ ^]^ *Drosophila*,^[^ ^209^ ^]^ zebrafish,^[^ ^104^ ^]^ *Xenopus* tadpole,^[^ ^152^ ^]^ *Xenopus laevis*,^[^ ^33^ ^]^ and mouse/rat.^[^ ^37^, ^80^, ^95^, ^118^, ^210^ ^]^ Although the application of nanotransducers in behavioral control has received great attention, the essence is mostly to use nanotransducers to manipulate [Ca^2+^]_i_ to elicit neural responses in target regions. Therefore, we present the main research methods in this field through several examples and summarize advanced works in **Table** **2** to illustrate the current state of the study. As an example, Bansal et al. first applied quasi‐continuous wave (CW) NIR light for UCNP‐based optogenetic manipulation of *C. elegans*.^[^ ^59^ ^]^ The mechanosensory neurons were genetically engineered with ChR2 channels, and UCNPs were taken up into the alimentary canal. The upconverted blue light was converted into a “touch” signal to activate mechanosensory neurons and elicit a reversal response consisting of a sudden stop, an omega turn, and a change in movement direction to avoid the light (**Figure** **15**A). Unlike lower animals, the neural circuits that govern diverse behaviors in mice and rats are considerably more sophisticated. To circumvent the BBB permeability issue and achieve a high local concentration close to the injection site, most studies deliver nanotransducers directly to the specific brain regions via intraparenchymal injection. The Anikeeva team applied AMF stimulation to release clozapine N‐oxide (CNO) from thermally responsive magnetoliposomes for chemogenetic activation of mice.^[^ ^145^ ^]^ CNO was gradually released from DPPC liposomes in response to an AMF to stimulate the hM3D(Gq), a designer receptor exclusively activated by designer drug (DREADD) engaging the Gq signaling pathway. To shape the behavior of freely moving mice, neurons in the ventral tegmental area (VTA) were first virally infected to express hM3D(Gq), followed by the local injection of magnetoliposomes. When mice were exposed to an AMF, CNO was released in the VTA and significantly enhanced their swimming (Figure 15B).

**Table 2:** Summary of recently reported nanotransducers in animal behaviors control.

Animal species	Neuron type/region	Ion channel	Output stimuli by nanotransducers	Behavior	Ref.
*C. elegans*	All sensory neurons in the amphid region	Unknown	Magnetothermal heating	Backward locomotion	[37]
	Mechanosensory neurons	Exogenous ChR2 channel	NIR light	Backward locomotion	[207]
*Hydra vulgaris*	Whole body column	Unknown	ROS	Enhance the contraction events	[226]
*Drosophila*	Unknown in the head	Exogenous TRPA1‐A channel	Magnetothermal heating	Wing extension	[145]
Zebrafish	Unknown in the yolk of zebrafish embryos	Endogenous L‐type channel	Electricity	Coiling behavior	[227]
*Xenopus laevis*	Under the thigh	Endogenous TRP channel	ROS and heat	Paw twitch	[210]
*Xenopus* tadpole	Cerebral ventricle	Endogenous IP_3_R	H_2_O_2_	Tail swaying	[152]
Mouse/rat	Dopaminergic neurons in the ventral tegmental area (VTA) region (≈4–6.6 mm under the scalp)	Exogenous TRPV1, ChR2, hM3D(Gq) channels	Magnetothermal heating, photothermal heating, NIR light, clozapine N‐oxide	Dopamine release for conditioning their location preference and increased mobility in a forced swim test	[46, 80, 86, 112, 205]
	Dorsal striatum region (≈3 mm under scalp)	Exogenous TRPV1, ChR2, TRPV4 channels, mechanosensitive ion channel	Magnetothermal heating, NIR light, magnetic force, mechanical force	Unilaterally rotation around the body axis	[80, 86, 95, 210]
	Visual cortex (≈1 mm under the scalp)	Exogenous ChR2 channel	NIR light	Animal training to obtain a water reward in a dual‐port chamber	[86]
	Motor cortex (≈0.55 mm under the scalp)	Mechanosensitive ion channel, VGCC, ChR2	Ultrasound, non‐Faradaic current, light	Limb movement	[68, 95, 118]
	The subthalamic nucleus (≈4.5 mm under the scalp)	Exogenous TRPV1 channels or endogenous VGCC	Magnetothermal heating, electrical stimulation	Reversed the motor deficits in the PD model; increased running speed in the Catwalk test	[112, 205]
	Nucleus accumbens (≈4.5 mm under the scalp)	Dopamine receptor D1 (DRD1)	DRD1 SFK‐38 393	Increased social preference	[46]
	Secondary motor cortex (≈2 mm under the scalp)	Exogenous TRPV1, Piezo1 channels, membrane depolarization	Magnetothermal heating, photothermal heating, magnetic force	Unilateral circling behavior	[46, 80, 86, 210]
	The ridge between dorsal and ventral striatum (≈4.1 mm under the scalp)	Exogenous TRPV1, TRPV4 channels, mechanosensitive ion channel	Magnetothermal heating, magnetic force, ultrasound, mechanical force	Freeze of gait	[80, 95, 210]
	Hippocampus (≈1.5 mm under the scalp)	Exogenous ChR2 channel	NIR light	Memory recall	[112]

### 3.7 [Ca2+]i‐Controlled Secretion of Bioactive Mediators

**Figure 16:**
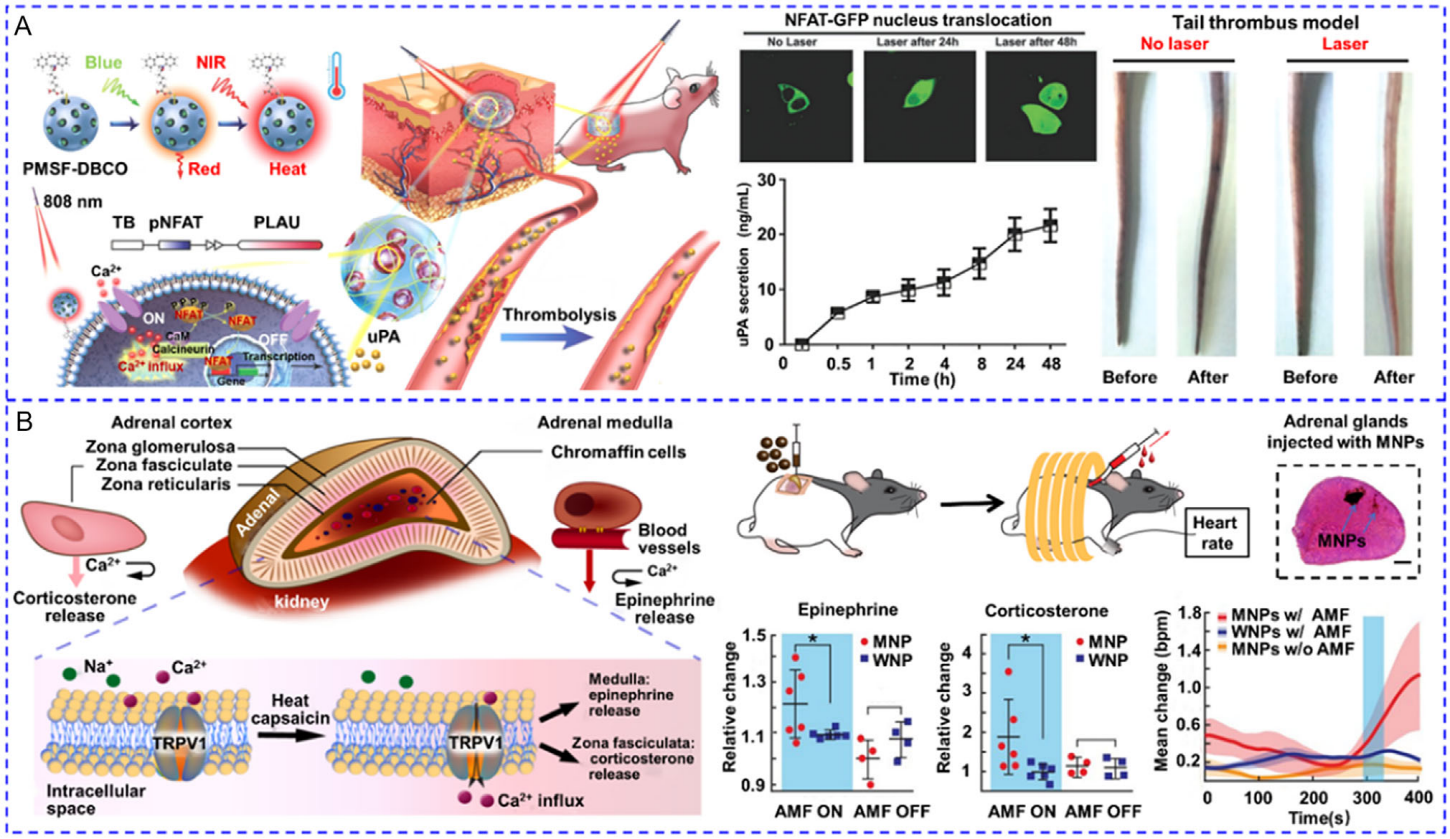
A) Engineered cell‐based hydrogel (ECH) for thrombolysis therapy. After NIR irradiation, NFAT‐GFP translocated to the nucleus, and secretion of uPA increased in TRPV1‐overexpressed HEK293T cells. The tail thrombus area dramatically reduced after the treatment. Reproduced with permission.^[^ ^32^ ^]^ Copyright 2019, Wiley. B) Ca^2+^ regulated the release of corticosterone and epinephrine from the adrenal cortex and medulla. The corticosterone and epinephrine levels in the blood increased, and the heart beat more rapidly upon AMF stimulation. Reproduced with permission.^[^ ^213^ ^]^ Copyright 2020, American Association for the Advancement of Science.

Ca^2+^‐triggered signaling cascades enable cells to adapt to environmental changes by controlling the secretion of bioactive mediators, such as hormones and functional proteins, into the bloodstream, which functions in the regulation of physiological activities.^[^ ^211^, ^212^ ^]^ Inspired by this physiological property, some groups have constructed engineered cell implants with nanotransducers that can be precisely regulated by external stimuli to achieve switchable secretion of bioactive mediators. Cell functioning was realized by introducing exogenous genetic circuits based on Ca^2+^/calcineurin/NFAT pathways. Briefly, a rise in [Ca^2+^]_i_ activates Ca^2+^‐sensitive calcineurin to dephosphorylate the NFAT transcription factor. NFAT then translocates to the nucleus, binds to the NFAT response element, and initiates the transcription of downstream target genes. In 2012, Stanely et al. developed a magnetothermal heating‐controlled proinsulin release biosystem by activating TRPV1 channels in bioengineered PC‐12 cell implants in nude mice.^[^ ^35^ ^]^ Increased [Ca^2+^]_i_ initiated the secretion of proinsulin and regulated blood glucose concentration. Zhang et al. designed a photoactivable engineered cell‐based hydrogel (ECH) to express urokinase plasminogen activator (uPA) for the treatment of thrombosis.^[^ ^32^ ^]^ In their design, the polymer dots hybridized mesoporous silica framework could generate a photothermal effect in response to NIR illumination and open TRPV1 channels for Ca^2+^ influx. Increased cytosolic Ca^2+^ acted as a secondary switch to stimulate the production of urokinase plasminogen activator (uPA). NIR light irradiation enabled ECH to secret uPA in a controlled and sustained manner. In the mouse tail thrombus model, four cycles of repeated irradiation for four days of the implanted ECH in mice significantly enhanced the thrombolysis therapeutic effects, as evidenced by a 90% reduction in the tail thrombus area (**Figure** **16**A).

Although it has been established that these cell implants efficiently and controllably produce and release bioactive mediators, future in vivo and clinical applications must account for the risk of immune rejection and carcinogenesis of exogenous cells. Nevertheless, there is no doubt that the manipulation of [Ca^2+^]_i_ to control the secretion of hormones from the patient's endocrine glands is a feasible technique for treating certain diseases. This inference was supported by the research of the Anikeeva team.^[^ ^213^ ^]^ Immunohistochemistry demonstrated that over 20% of rat adrenal cells overexpressed TRPV1 channels, allowing for magnetothermal regulation of Ca^2+^ influx into adrenal cells. In vivo studies showed that in situ injections of MNPs into the adrenal gland could considerably increase blood corticosterone and epinephrine concentrations via magnetothermal heating, and induced an increase in the heart rate (Figure 16B).

### 3.8 Others

**Figure 17:**
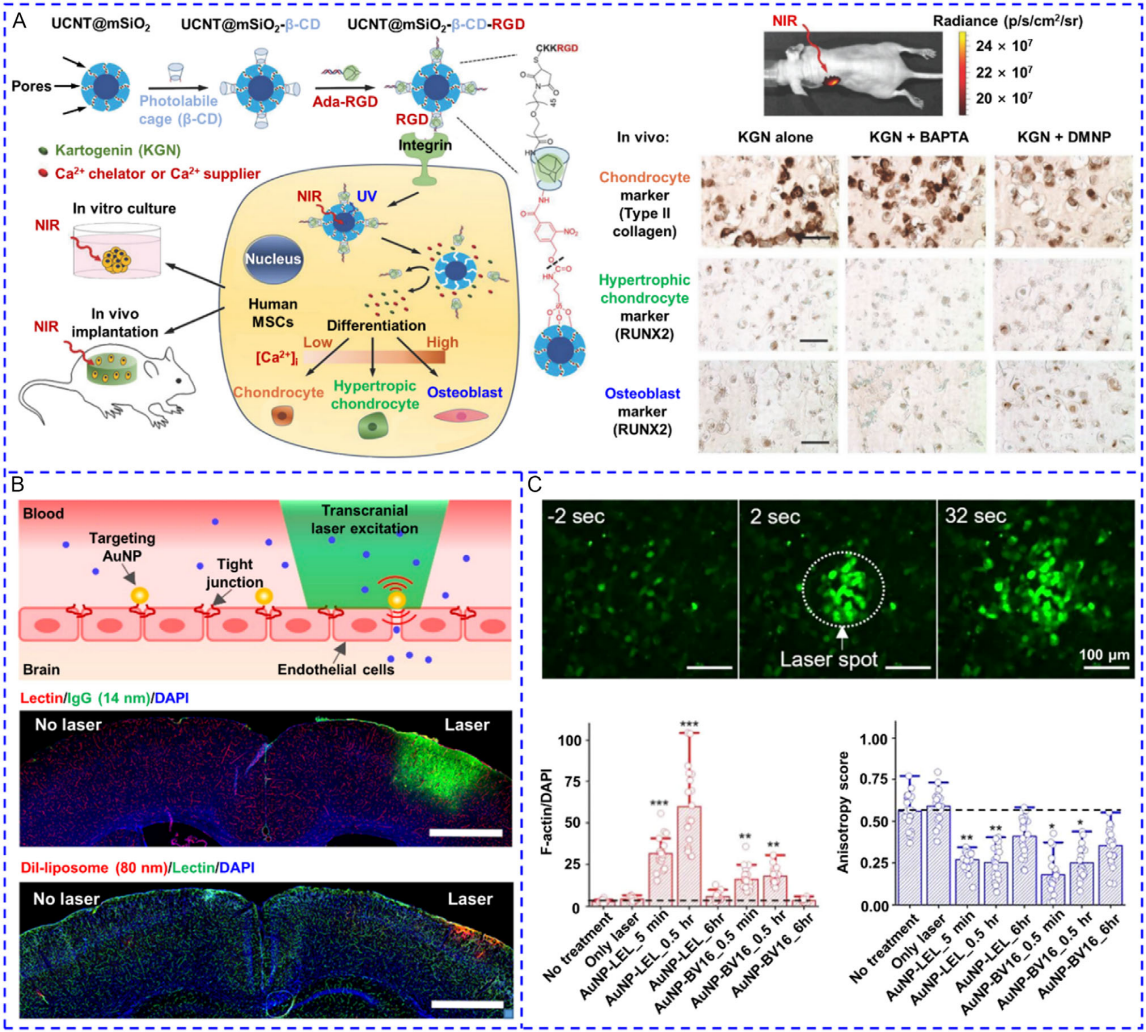
A) Utilization of UCT@mSiO_2_‐β‐CD‐RGD for the delivery of kartogenin (KGN) and Ca^2+^ chelator/supplier and control of the differentiation of MSCs into three lineages. Immunochemistry images show the expression of markers of chondrocytes, hypertrophic chondrocytes, and osteoblasts from in vivo implants. Reproduced with permission.^[^ ^214^ ^]^ Copyright 2018, Wiley. B) Irradiation of Au NPs by transcranial laser could transiently open the BBB by targeting tight junctions of endothelial cells. Lectin and liposome as models can pass BBB after laser irradiation. Reproduced with permission.^[^ ^216^ ^]^ Copyright 2021, American Chemical Society. C) Laser stimulation of AuNPs‐triggered Ca^2+^ responses in endothelial cells and affected fluorescent intensity and anisotropy score of F‐actin. Reproduced with permission.^[^ ^217^ ^]^ Copyright 2023, Royal Society of Chemistry.

Numerous efforts have been made to regulate Ca^2+^ signaling for the directional differentiation of stem cells. In this section, successful examples are presented to reveal the advanced research progress in the field. For instance, Ma et al. utilized polydopamine‐coated piezoelectric nylon‐11 NPs to stimulate the osteogenic differentiation of dental pulp stem cells (DPSCs).^[^ ^107^ ^]^ Results show that highly biocompatible nylon‐11 NPs were efficiently endocytosed by dental pulp stem cells. Ca^2+^ flowed into stem cells after ultrasound treatment to accelerate their osteogenic differentiation, as evidenced by the formation of calcium mineralized nodules and upregulated expression of osteopontin. More subtly, Kang et al. designed a photocleavable nanocarrier to precisely control stem cell differentiation into different lineages by decreasing or increasing [Ca^2+^]_i_.^[^ ^214^ ^]^ A nanocarrier was prepared by covering UCNPs with a mesoporous silica shell, and the surface pores were sealed with photolabile caps after cargo loading to prevent premature leakage. Kartogenin (KGN, a chondroprotective agent) and a chemical Ca^2+^ modulator, i.e., a Ca^2+^ chelator or supplier, were co‐loaded into nanocarriers to regulate stem cell differentiation. After taken up by mesenchymal stem cells, the combined release of KGN and Ca^2+^ chelator (i.e., BAPTA‐AM) after photo‐uncaging of nanocarriers induced mesenchymal stem cells differentiation into chondrocytes (**Figure** **17**A). Conversely, the release of KGN and the Ca^2+^ supplier (i.e., DMNP‐EDTA‐Ca^2+^) promoted osteoblast differentiation.

The blood–brain barrier (BBB), formed by a border of endothelial cells, serves as a highly selective semipermeable interface between the bloodstream and brain parenchyma for the exchange of essential nutrients while restricting the passage of majority of hydrophilic and large molecules into the central nervous system (CNS).^[^ ^215^ ^]^ Despite its protective role, BBB greatly impedes the efficient transport of therapeutic medications to the brain for CNS diseases. Hence, a transient increase in BBB permeability without damaging vascular structures and brain parenchyma is in high demand. To date, only a few research has been conducted on manipulating [Ca^2+^]_i_ with nanotransducers to increase the permeability of the BBB. In 2021, the Qin team discovered that the transcranial picosecond laser irradiation on Au NPs targeting tight junctions of endothelial cells could controllably and reversibly modulate BBB permeability (Figure 17B).^[^ ^216^ ^]^ Later, their further research revealed that Au NPs generated both tiny mechanical and thermal perturbation in response to a transcranial picosecond laser and caused a sharp increase in [Ca^2+^]_i_.^[^ ^217^ ^]^ Subsequently, Ca^2+^ waves spread to adjacent endothelial cells to expand the BBB opening region (Figure 17C). Meanwhile, increased [Ca^2+^]_i_ triggered actin polymerization and promoted ERK1/2 phosphorylation, which in turn activated the cytoskeleton to increase paracellular permeability. This technology has been demonstrated to enhance the delivery of paclitaxel, an anticancer agent that failed in early‐phase clinical trials for glioblastoma treatment due to its inability to penetrate BBB, to effectively treat glioblastoma.^[^ ^218^ ^]^ Noticeably, modulation of [Ca^2+^]_i_ with nanotransducers to enhance BBB permeability is still in its exploratory stage. Particularly, the transport of nanotransducers to the target region, the efficient stimulation of nanotransducers for the propagation of intercellular Ca^2+^ waves, and the investigation of bystander effects during Ca^2+^ signaling transduction need to be further investigated.

## 4 Conclusions and Perspectives

The emergence of nanotransducers has revolutionized the field of Ca^2+^ signaling. Unique properties of nanotransducers including the nanoscale size with a high surface‐to‐volume ratio of nanomaterials, versatile energy conversion capability, and catalytic activity of nanoplatforms, permit design options for [Ca^2+^]_i_ manipulation with high controllability, precision, and spatiotemporal resolution, which have greatly facilitated the comprehension of the mechanism of Ca^2+^ signaling regulation. In addition, it has contributed to the identification of the role of Ca^2+^ signaling in the regulation of physiological activities and the development of clinical therapies based on Ca^2+^ signaling. In this review, we have surveyed the current state of nanotransducers for regulating Ca^2+^ signaling and their biomedical applications. The coupling of their chemical and structural features with stimulated Ca^2+^ channels is highlighted, the manipulation efficiency and application prospect are evaluated, and the activated downstream genes and neural circuits and their induced biological responses are discussed.

Despite recent advances in the development of emerging nanotransducers for manipulating [Ca^2+^]_i_, there are still obstacles that need to be overcome. Existing scientific concerns and proposals for the development of nanotransducers in subjugating the bottlenecks of Ca^2+^ signaling fields are shown below. 1) Several ROS, i.e., H_2_O_2_ and ^1^O_2_, have been observed to directly stimulate Ca^2+^ channels such as TRPV1 and TRPV4 for Ca^2+^ influx.^[^ ^219^, ^220^ ^]^ However, the capability and efficiency of other potent ROS, such as hydroxyl radical, and superoxide radical, to manipulate Ca^2+^ has been seldom explored and remains to be evaluated. 2) Biomimetic functional nanomaterials, such as exosomes, and outer membrane vesicles, are promising platforms for delivering chemical modulators due to their enhanced bioavailability and biosafety.^[^ ^221^, ^222^ ^]^ However, the relevant researches in this field are still lacking. 3) The primary strategy of Ca^2+^ manipulation is based on the activation of Ca^2+^ channels, which often requires viral infection to overexpress these channels. However, viral infection typically takes 2–8 weeks and is often accompanied by biosafety risks, which severely restricts the therapeutic application of nanotransducers. In addition, several cation channels, such as ChR2 and TRP, are highly permeable to other cations (i.e., H^+^, Na^+^), which may result in unwanted ion interference.^[^ ^23^ ^]^ Therefore, it is highly desirable to develop more practical methods for inserting Ca^2+^ channels with high Ca^2+^ selectivity into the cell membrane. The rational design of artificial transmembrane ion channels based on synthetic chemical compounds with excellent ion selectivity and easy introduction into cell lipid layers holds great promise. Very recently, our team discovered a single‐chain random heteropolymer‐derived ion channel for highly selective K^+^ transport.^[^ ^223^ ^]^ Polymers were easily and spontaneously inserted into the liposome membrane and demonstrated high membrane compatibility, preferable K^+^ recognition, and light‐gated transporting. Being inspired, the development of artificial Ca^2+^‐selective transmembrane ion channels with a stimuli‐responsive switch is a viable alternative to viral infection. 4) Calcium‐based nanomaterials contain extremely high Ca^2+^ density and are ideally suited for long‐term [Ca^2+^]_i_ modulation. However, their direct use to release Ca^2+^ in acidic lysosomes within cells is uncontrollable. One solution is to coat calcium‐based nanomaterials with polymer or lipid shells whose permeability to H^+^ can be reversibly adjusted in response to temperature. Another approach is to combine the photoacid with calcium‐based nanomaterials so that the Ca^2+^ release profile can be directly controlled by light irradiation. 5) As can be seen in the manuscript, the majority of nanotransducers were designed to increase [Ca^2+^]_i_, and only a few nanotransducers are capable of causing a decrease in [Ca^2+^]_i_. However, to the best of our knowledge, no such a nanotransducer can reversibly regulate [Ca^2+^]_i_. Thus, the development of sponge‐like nanotranducers which can release and absorb Ca^2+^ under different conditions for flexible regulation [Ca^2+^]_i_ is a foremost direction in the future. 6) Most nanotransducers are based on inorganic materials, and their high stability in tissues especially deep in the brain makes their long‐term safety and toxicity profiles questionable. Many natural biodegradable materials, including melanin NPs and bacteria, which have been widely used for cancer photothermal therapy, can also be used as potential candidates for the photothermal activation of TRP channels. 7) Tissue penetration depth of NIR light is severely limited and is most suitable for 2D cells and superficial tissues. Ultrasound‐induced organic mechanoluminescence materials can provide a light source deep in the tissue due to the excellent tissue penetration depth of acoustic waves. Thus, a more mechanoluminescent nanotransducer should be designed for future study. 8) The short temporal response after applying the external stimuli is always the pursued goal for designing new [Ca^2+^]_i_ manipulating nanotransducers, which is determined by both the energy conversion rate of the nanotransducer and the Ca^2+^ channel response threshold. For example, compared to the short temporal response (i.e., ≈ms) mediated by light or photothermal heating, magnetothermal heating is relatively slow so that the temporal response is ca. a few to tens of seconds (Table 1). One solution is to develop MNPs with higher conversion efficiency, which is limited by the current synthetic technique and the intrinsic properties of materials. Another solution is to couple MNPs with heat rate‐sensitive Ca^2+^ channels, instead of threshold thermoreceptors, which has been much less studied.^[^ ^209^ ^]^ Therefore, how to reduce the response time is still worth further exploration. 9) Nanomotors as mobile electric converters were driven by the magnetic field or light for [Ca^2+^]_i_ manipulation in this review. In fact, more potentials of nanomotors can be further explored. For example, nanomotors can be designed as mobile converters to produce other stimuli, such as heat, mechanical force, and chemical stimuli. More importantly, the motility of nanomotors allows the selective manipulation of multiple cells with a small number of nanomotors, which has important academic value for the study of Ca^2+^ signaling. Therefore, the design of the nanomotors‐based nanotransducers should be paid attention to in the follow‐up development.

## Conflict of Interest

The authors declare no conflict of interest.
